# Towards Right Answer for the Right Reasons in Global Land Carbon Sink Estimates

**DOI:** 10.1111/gcb.70840

**Published:** 2026-04-07

**Authors:** Helin Zhang, Youngryel Ryu, Sungchan Jeong

**Affiliations:** ^1^ Research Institute of Agriculture and Life Sciences Seoul National University Seoul Republic of Korea; ^2^ Department of Landscape Architecture and Rural Systems Engineering Seoul National University Seoul Republic of Korea; ^3^ SNU Energy Initiatives Seoul National University Seoul Republic of Korea

**Keywords:** FLUXNET, land carbon sink, net ecosystem exchange, R_eco_‐GPP relationship, uncertainties

## Abstract

Quantifying the global land carbon sink is essential for understanding carbon‐climate feedbacks and developing effective mitigation strategies. Despite decades of research using eddy covariance, remote sensing, and atmospheric observations, carbon sink estimates remain highly uncertain across different approaches. The key challenge is to obtain the right global land carbon sink estimates for the right reasons. In this review, we first provide an overview of current methodologies for estimating terrestrial carbon sinks, including FLUXNET observations, top‐down atmospheric inversions, and bottom‐up approaches. We then leverage insights learned from FLUXNET to evaluate the observed range of annual net ecosystem exchange (NEE), gross primary production (GPP)–ecosystem respiration (R_eco_), and GPP–evapotranspiration (ET) relationships, and assess whether current models capture these observational constraints. Because FLUXNET is widely used as a benchmark for evaluation and as training data for model calibration, we also identify opportunities to strengthen the utility of FLUXNET for global land carbon sink inference.

## Graphical abstract

We review the main approaches used to estimate the global land carbon sink. Using evidence from FLUXNET, we show that improving these estimates requires not only matching the total land carbon sink, but also correctly capturing the links among carbon uptake, respiration, and water loss. We further discuss the limitations of FLUXNET and how it can be better used to improve future global carbon sink estimates.

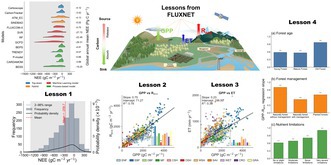

## 1 Introduction

Global land carbon sink estimates are highly uncertain. The Global Carbon Project, the most widely used dataset of global carbon cycles, provides global land carbon sink estimates of 3.2 ± 0.9 PgC year^−1^ between 2014 and 2023 (Friedlingstein et al. 2025). A recent study, however, suggested a smaller global land carbon sink of 2.7 ± 0.9 PgC year^−1^ for 2014–2023 after accounting for evolving land‐cover change and a correction for lateral carbon export (Friedlingstein et al. 2026). Additionally, Randerson et al. (2025) proposed a much weaker global land carbon sink estimate of 0.8 ± 0.7 PgC year^−1^ between 2000 and 2019 by adjusting fossil fuel emissions and ocean carbon uptake. How can these contrasting estimates be reconciled?

To obtain the right answer for the right reasons in global land carbon sink estimates, we advocate leveraging ground observations of net ecosystem exchange (NEE, with negative values indicating net CO_2_ uptake and positive values indicating net release to the atmosphere) measured through a global network of eddy covariance towers, FLUXNET (Baldocchi 2008; Jung et al. 2020; Pastorello et al. 2020). FLUXNET data do not cover the global land surface; they underrepresent important carbon hotspots such as tropical forests and disturbed or fire‐affected areas, and fail to adequately span the full aridity gradient across global dryland ecosystems (Sulkava et al. 2011; Papale et al. 2015; Chu et al. 2017; Smith et al. 2019). In addition, eddy covariance measurements capture CO_2_ emissions from fires and land‐cover change only within the tower footprint, and lateral carbon flows remain undetected. However, FLUXNET is the only tool that directly measures NEE continuously at the ecosystem scale, whereas there is no direct method to measure net biome productivity (NBP) (Kutsch et al. 2010; Kirschbaum et al. 2019). Although the global land carbon sink is ultimately quantified as NBP, NEE constitutes its core component. Evaluating model performance in simulating NEE is therefore a necessary first step, as biases in modeled NEE will propagate into NBP estimates. A series of key lessons learned from FLUXNET—which we will review later—offers an excellent framework to evaluate whether different models can reproduce these key lessons.

Land carbon sink estimation methods generally fall into two main categories: top‐down and bottom‐up approaches (Crisp et al. 2022). Top‐down approaches estimate regional or global carbon budgets by inversely modeling atmospheric CO_2_ observations, often relying on atmospheric chemistry transport models and satellite or ground‐based measurements of CO_2_ concentrations (Fung et al. 1983; Tans et al. 1990; Peiro et al. 2022). They provide integrative, large‐scale constraints but offer less insight into local ecological processes (Miller et al. 2007; Crisp et al. 2012). Bottom‐up approaches aggregate local information to larger scales using inventory‐based estimates (Birdsey et al. 1993; Houghton 2003; Ciais, Paris, et al. 2010), FLUXNET‐based machine learning (ML) models (Papale and Valentini 2003; Xiao et al. 2010; Jung et al. 2020), bookkeeping models (Houghton et al. 1983; Aguiar et al. 2012; Hansis et al. 2015), or process‐based models (Sellers et al. 1997; Sitch et al. 2003; Ryu et al. 2011). Although bottom‐up approaches provide detailed, process‐based information at finer scales, uncertainties might accumulate during upscaling to regional or global levels (Ciais et al. 2014; Schimel et al. 2015; Crisp et al. 2022). Each method thus frames land sink inference around different advantages and assumptions, providing different “reasons” for the resulting estimates.

**FIGURE 1:**
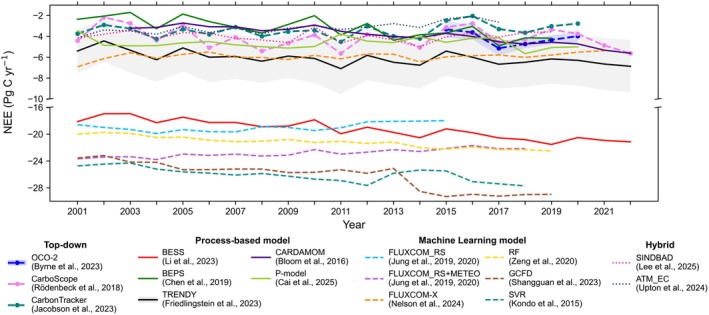
Time series of global annual net ecosystem exchange (NEE) from multiple datasets. Following Nelson et al. (2024), OCO‐2 and CarboScope were fire‐adjusted by subtracting Global Fire Emissions Database version 4.1 (Randerson et al. 2017). For OCO‐2 and TRENDY, solid lines show the ensemble mean and shaded bands show inter‐model spread (±1 SD): Blue for OCO‐2 and grey for TRENDY.

Large, method‐dependent discrepancies currently obscure the magnitude of the global NEE estimates (Piao et al. 2020; Heinrich et al. 2021; Upton et al. 2024). Figure 1 highlights pronounced gaps between different models, with published global NEE ranging from modest to very strong sinks. Such divergence between top‐down and bottom‐up estimates has been reported across both global and regional cases (Kondo et al. 2015; Steinkamp et al. 2017). Yet the spread among bottom‐up models is similarly large. For example, the Biosphere‐atmosphere Exchange Process Simulator (BEPS) (Chen et al. 2019) estimated global NEE as −3.18 ± 0.91 PgC year^−1^, whereas several FLUXNET‐based ML models suggested global NEE values approaching or exceeding −20 PgC year^−1^ (Kondo et al. 2015; Jung et al. 2020; Zeng et al. 2020). These discrepancies are also evident in spatial patterns: mean annual NEE distributions differ markedly among products, particularly in tropical regions (Figure S1), and temporal trends show divergent magnitudes and even opposite signs across different products (Figure S2). Consequently, global NEE estimates span a wide range, yielding different “answers” for the global land carbon sink.

To reconcile the large uncertainties and discrepancies among different global NEE estimates, several critical questions emerge: What insights do FLUXNET observations provide about the underlying mechanisms and magnitude of the global land carbon sink (the “right reasons”)? Do current models align with these observationally‐informed insights? Finally, how can we leverage these “right reasons” to arrive at a more accurate and realistic estimation (the “right answer”) of the global land carbon sink? To tackle these issues, this review first provides an overview of the major approaches used to estimate the global land carbon sink, covering top‐down atmospheric inversions and bottom‐up methods. We then explore what can be learned from FLUXNET about getting the right answer for the right reasons, focusing on the observed range of annual NEE, the coupling between gross primary production (GPP) and ecosystem respiration (R_eco_), and carbon‐water coupling in GPP–Evapotranspiration (ET) relationships, together with the effects of forest age, forest management, and nutrient limitations on the R_eco_‐GPP slope. Finally, we synthesized key challenges to strengthen the utility of FLUXNET for robust global NEE estimates.

## 2 Methods for Estimating Land Carbon Sinks: What Tools are we Using?

### 2.1 FLUXNET

If satellites give us a bird's‐eye view of the Earth, the global network of eddy covariance towers monitors the “breathing” of the terrestrial biosphere (Baldocchi et al. 2001). Baldocchi (2025) provides a historical perspective on how the global flux‐tower network has developed over the past four decades. By directly measuring exchanges of carbon, water, and energy across diverse ecosystems, this evolving FLUXNET network provides essential ground truth for understanding and constraining the land carbon sink (Baldocchi 2014; Keenan and Williams 2018). FLUXNET was not designed to maximize spatiotemporal coverage and thus cannot directly quantify the global land carbon sink (Sulkava et al. 2011; Schimel et al. 2015; Chu et al. 2017). Yet it provides an observational benchmark for evaluating whether models derive their global NEE estimates for the right reasons.

### 2.2 Top‐Down

Top‐down approaches infer surface CO_2_ fluxes by assimilating atmospheric CO_2_ concentration data into transport models that simulate how emissions and sinks influence the observed distribution of CO_2_ (Bolin and Keeling 1963; Fung et al. 1983; Ciais, Rayner, et al. 2010). Top‐down approaches consist of three key components: atmospheric CO_2_ observations, atmospheric chemistry transport models, and inversion algorithms (Ciais, Rayner, et al. 2010; Thompson et al. 2022). Ground‐based networks or satellite observations provide spatiotemporal constraints on atmospheric CO_2_, these constraints are linked to surface fluxes via atmospheric transport (Enting and Mansbridge 1989; Baker et al. 2006; Thompson et al. 2022). Using statistical inversion techniques such as Bayesian optimization or ensemble‐based methods, these systems iteratively adjust flux estimates to reconcile model outputs with observed atmospheric concentrations (Peylin et al. 2013; Thompson et al. 2022).

Several operational inversion systems have been developed to provide regular carbon flux estimates at global and regional scales. Prominent examples include CarbonTracker from NOAA (Peters et al. 2007; van der Velde et al. 2018; Jacobson et al. 2023), CAMS CO_2_ Flux system operated by ECMWF (Agustí‐Panareda et al. 2014, 2016), NASA CMS‐Flux framework (Liu et al. 2018, 2021), Global Carbon Assimilation System GCAS (Zhang et al. 2015; Jiang et al. 2021), CarboScope maintained by BGC‐Jena (Rödenbeck et al. 2018; Munassar et al. 2025), and MIROC4‐ACTM inversion system from JAMSTEC (Chandra et al. 2022). These systems differ in their spatial resolution, inversion algorithms, and input datasets, but all aim to reduce uncertainties in carbon budget estimates by leveraging the increasing volume and quality of atmospheric CO_2_ observations. Complementing these CO_2_‐focused inversions, O_2_‐based methods exploiting atmospheric oxygen‐to‐nitrogen ratios (O_2_/N_2_) leverage distinct stoichiometric fingerprints to construct atmospheric potential oxygen tracers, enabling independent separation of terrestrial versus oceanic carbon uptake (Keeling and Shertz 1992; Welp et al. 2011).

Despite their critical role in global carbon sink estimation, top‐down estimates remain subject to multiple sources of uncertainty that limit their accuracy and resolution (Wunch et al. 2017; Stanevich et al. 2020; Peiro et al. 2022). Major error sources include heterogeneous spatial coverage of ground‐based monitoring stations (Peylin et al. 2013; Basu et al. 2018; Crowell et al. 2019), systematic measurement biases affecting satellite retrievals (Wunch et al. 2017; Massie et al. 2021; Das et al. 2025), incomplete representation of vertical mixing and boundary layer dynamics within transport models (Baker et al. 2006; Basu et al. 2018), and sensitivity to assumed prior flux distributions (Chandra et al. 2022). These challenges have prompted coordinated inversion model intercomparison projects (MIPs), such as the OCO‐2 MIP (Peiro et al. 2022; Byrne et al. 2023). Overall, these uncertainties highlight the need for ongoing improvements in observational networks, transport modeling, and inversion methodologies to enhance the robustness of top‐down carbon flux assessments.

### 2.3 Bottom‐Up

Bottom‐up approaches estimate carbon sinks by aggregating local‐scale information to regional and global scales (Intergovernmental Panel on Climate Change (IPCC) 2014; Friedlingstein et al. 2023). Unlike top‐down approaches, bottom‐up methods explicitly focus on quantifying ecosystem carbon cycling through various pathways, from direct measurements of carbon stocks (above‐ground biomass, below‐ground biomass, dead wood, litter and soil carbon) and fluxes (photosynthesis, respiration, decomposition) (Chapin et al. 2006; Baldocchi 2008). These methods encompass both process‐based models that simulate biogeochemical mechanisms and machine learning approaches that capture statistical relationships from observational data. While the depth of mechanistic insight varies among different bottom‐up approaches, they collectively contribute to understanding “where carbon sinks originate, how they change, and where they are going” (Kondo et al. 2020; Winkler et al. 2023). In this review, we focused on process‐based models and FLUXNET‐based ML methods, with detailed analysis provided in Section 3. For brevity, we do not review other bottom‐up frameworks and complementary tracers in depth (e.g., inventories, bookkeeping, and additional observational constraints), focusing instead on process‐based models and FLUXNET‐trained ML products.

Process‐based models are mechanistic models that incorporate physical and physiological processes to simulate carbon, water, and energy cycles and their associated biomass and soil carbon dynamics (Sitch et al. 2003; Bloom et al. 2016; Yuan et al. 2024). These models explicitly simulate the processes controlling terrestrial carbon dynamics driven by environmental factors, land cover change, and vegetation structural dynamics (Sitch et al. 2015; Chen et al. 2019; Ruehr et al. 2023). The TRENDY dataset employs ensemble simulations from different dynamic global vegetation models (DGVMs) to assess the natural land carbon sink contribution to the global carbon budget (Sitch et al. 2008, 2015; Friedlingstein et al. 2023). Additionally, other widely used process‐based models include the Breathing Earth System Simulator (BESS) (Ryu et al. 2011; Jiang and Ryu 2016; Li, Ryu, et al. 2023), BEPS (Ju et al. 2006; Chen et al. 2019), and the P‐model (Stocker et al. 2020; Cai et al. 2025), as well as the CARbon DAta MOdel fraMework (CARDAMOM) that integrates process‐based modeling with data assimilation techniques (Bloom et al. 2016). A widely adopted approach for enhancing model performance involves incorporating an increasing array of known carbon cycle processes, along with improvements in the mathematical representations and parameterizations of these individual processes (Clark et al. 2011; Lawrence et al. 2019; Yuan et al. 2024). However, with advancing model development and increasing complexity, these approaches face growing challenges including large uncertainties in parameterization, insufficient observational constraints, and substantial differences in process representations across models (Fisher et al. 2018; Arora et al. 2020).

FLUXNET‐based ML models utilize machine learning approaches, combining multi‐source observational data (such as eddy covariance flux observations, meteorological data, and remote sensing data) to estimate carbon fluxes. The FLUXCOM series, including FLUXCOM‐RS, FLUXCOM‐RS + meteo, and the latest FLUXCOM‐X, represents the most widely used and typical example of data‐driven carbon flux datasets (Tramontana et al. 2016; Jung et al. 2019, 2020; Nelson et al. 2024). Other FLUXNET‐based ML products also achieve global terrestrial NEE estimation through diverse machine learning models, different forcing variables and input datasets (Kondo et al. 2015; Zeng et al. 2020; Shangguan et al. 2023). The advantage of FLUXNET‐based ML models lies in their direct learning from observational data and their ability to capture complex non‐linear relationships in the Earth system.

## 3 What Can we Learn From FLUXNET?

### 3.1 Ranges of Annual NEE

#### 3.1.1 Lessons from FLUXNET

**FIGURE 2:**
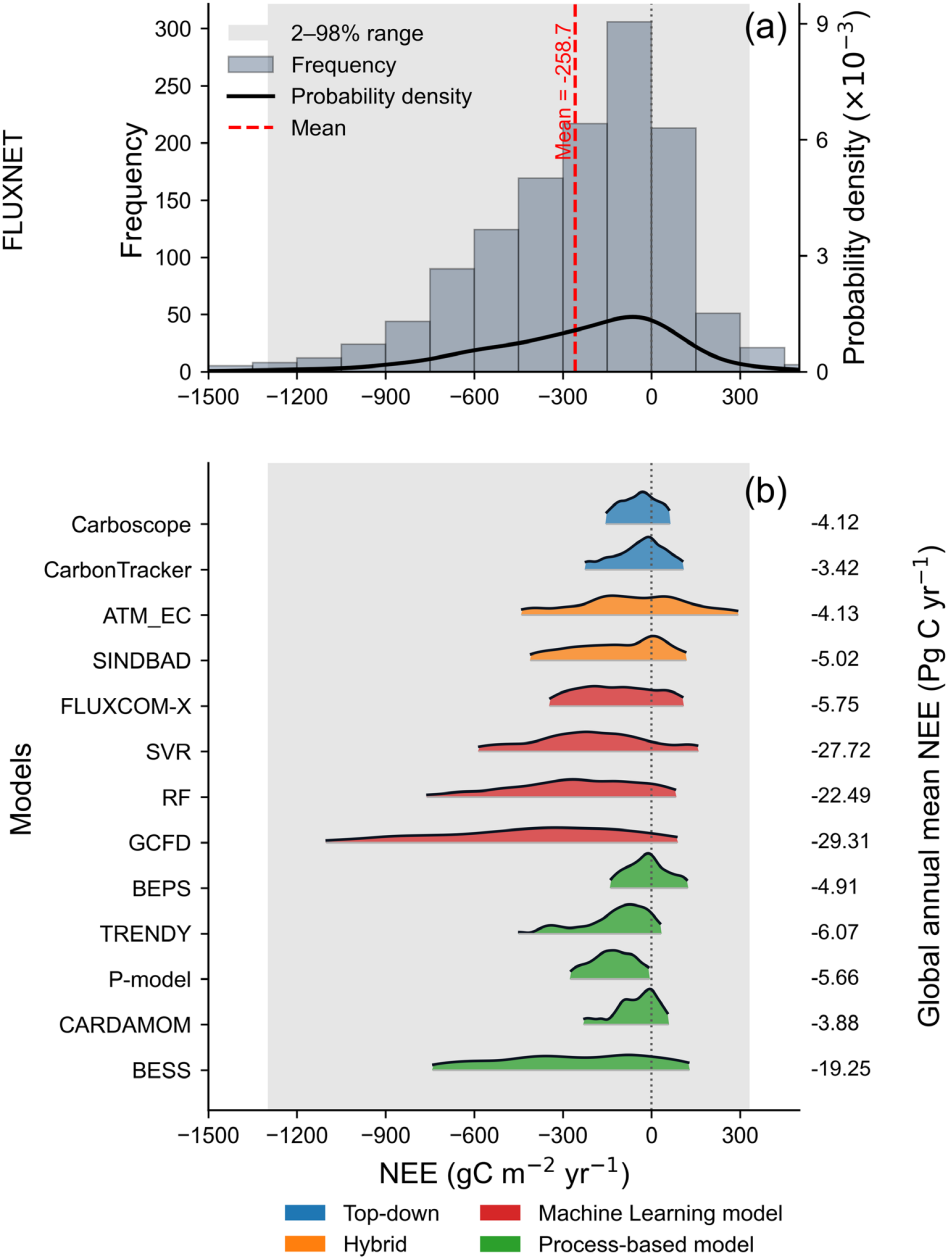
Annual NEE distributions across FLUXNET site‐years and model products. (a) Histogram (bars; left axis) and probability density function (solid line; right axis) of annual NEE across FLUXNET site‐year records (g C m^−2^ year^−1^). (b) Probability density functions of annual NEE from different model products after extracting model NEE for the same FLUXNET site‐years. Numbers on the right indicate global mean annual NEE (PgC year^−1^) from different models, averaged over the period shown in Figure 1. The light‐grey shading marks the FLUXNET NEE 2nd–98th percentile range to facilitate comparison across products.

FLUXNET observations have revealed the wide distribution of annual NEE (Baldocchi 2008, 2014). While most ecosystems function as net carbon sinks, the distribution of annual NEE extends from strong sinks (< −1000 gC m^−2^ year^−1^) to sources (> 300 gC m^−2^ year^−1^) (Falge et al. 2002; Baldocchi et al. 2018) (Figure 2a). The seminal analysis by Valentini et al. (2000) of 15 European forests documented a large NEE range of −660 to 90 gC m^−2^ year^−1^ spanning boreal to Mediterranean climates, establishing that southern warmer forests sequester substantially more carbon than northern cooler forests. Some studies demonstrated that annual NEE spans a broader range. For example, Abdalla et al. (2013) reported contrasts in annual NEE among co‐located ecosystems under the same climatic conditions in southeastern Ireland, with a young Sitka spruce plantation acting as a strong carbon sink (−904 gC m^−2^ year^−1^) due to enhanced carbon storage in woody biomass and soils, whereas nearby grassland (−212 gC m^−2^ year^−1^) and cropland (−189 gC m^−2^ year^−1^) showed much weaker net uptake. Subsequent studies further showed that this range widens under management and disturbance, with ecosystems switching from strong sinks to sustained sources during recovery phases (Lindauer et al. 2014; Lindroth et al. 2018). Dryland ecosystems also frequently alternate between functioning as C sinks and C sources in wet and dry years, with annual NEE ranging from −420 to +550 gC m^−2^ year^−1^ across 150 site‐years in dryland ecosystems of southwestern North America (Biederman et al. 2017). This broad distribution reflects the complex interplay of photosynthetic carbon uptake and respiratory carbon loss, modulated by factors including vegetation productivity, soil respiration, disturbance history, and climate variability (Valentini et al. 2000; Law et al. 2002; Luyssaert et al. 2007).

#### 3.1.2 Model Evaluations Against FLUXNET

However, various modeling approaches showed substantial differences in their ability to capture the range of observed annual NEE values (Figure 2b). Across products sampled at the same FLUXNET site‐years, top‐down model (Carboscope) and several process‐based models produced narrowly distributed, near‐zero NEE, whereas FLUXNET‐trained ML products exhibited broader distributions and stronger sinks. Notably, some products achieved plausible global totals while failing to reproduce the observed site‐level variability, exemplifying the risk of getting the “right answer” for the wrong reasons. Although the two hybrid models, ATM_EC and SINDBAD, had annual NEE peaks centered on weak carbon sinks close to zero (mean NEE of −62.62 and −77.98 gC m^−2^ year^−1^), they exhibited a much broader NEE range, with standard deviations of 119.13 and 112.97 gC m^−2^ year^−1^. This pattern can be attributed to their hybrid design, in which top‐down data constrain regional mean NEE, whereas bottom‐up information from flux towers and remote sensing provides finer spatial and temporal variability of the fluxes (Upton et al. 2024; Worden et al. 2025; Lee et al. 2025).

#### 3.1.3 Sources of Model‐Observation Divergence

The limited spread in top‐down NEE estimates largely indicates the coarse spatial resolutions and sparse coverage of the atmospheric CO_2_ concentration measurements used as constraints in the inversions. The top‐down models are constrained by sparse atmospheric CO_2_ measurements that integrate over large spatial domains, leading to smoothed flux patterns that prioritize consistency with atmospheric CO_2_ gradients over fine‐scale variability (Fung et al. 1983; Ciais, Paris, et al. 2010; Ciais et al. 2022). The resulting flux estimates become increasingly uncertain at smaller spatial scales (Schuh et al. 2013; Feng et al. 2019), with prior fluxes strongly influencing posterior results in data‐sparse regions and further dampening spatial heterogeneity (Feng et al. 2021; Philip et al. 2019). Importantly, top‐down inversions were designed to constrain large‐scale carbon budgets from atmospheric CO_2_ gradients, not to resolve fine‐scale ecosystem carbon dynamics. Atmospheric transport models further limit the ability to resolve fine‐scale CO_2_ gradients (Basu et al. 2018; Stanevich et al. 2020; Katharopoulos et al. 2023). Following Nelson et al. (2024), the top‐down flux products were fire‐adjusted by subtracting Global Fire Emissions Database version 4.1 (Randerson et al. 2017). However, other carbon loss pathways remain largely unconstrained, including lateral riverine export and carbon removal through harvest and grazing (Ciais et al. 2021; Byrne et al. 2023). If these processes were properly considered, the global top‐down NEE estimates would likely indicate a stronger carbon sink and exhibit a wider range of NEE values.

In process‐based models, the narrow, weak‐sink NEE distributions likely arose from three interrelated factors. First, most process‐based models rely on spin‐up procedures that assume carbon‐cycle steady state (Thornton and Rosenbloom 2005; Carvalhais et al. 2008; Li et al. 2011). However, terrestrial carbon cycles typically exist in a state of disequilibrium driven by internal ecosystem processes and external disturbances, rather than being constrained to a fixed equilibrium state (Luo and Weng 2011). Under steady‐state assumptions, carbon turnover time is biased low because carbon influx exceeds efflux in sink ecosystems, accelerating simulated carbon losses and narrowing the NEE distribution (Rodhe 1978). Model‐data fusion in eastern China confirmed that steady‐state assumptions underestimated carbon turnover time by ~29%, leading to a 4.8‐fold overestimation of NEE (Ge et al. 2019).

Second, spatial scale mismatches and meteorological forcing uncertainties contribute to the compressed NEE range in process‐based models. Eddy‐covariance fluxes represent sub‐kilometer to kilometer footprints (Chu et al. 2021; Kong et al. 2022), whereas DGVMs operate on much larger grid cells that smooth subgrid heterogeneity. In addition, gridded meteorological forcing datasets used to drive models may not capture local climate conditions at flux tower sites. Seiler et al. (2022) identified four potential sources of model‐observation discrepancy: spatial scale differences, meteorological forcing uncertainties, unrepresented disturbance history, and reduced data quality from gap‐filling. Even after restricting the FLUXNET benchmark to high‐quality measurements from sites where no disturbance occurred over the last 50 years, the modeled NEE range remained strongly compressed (model: −1.3 to 0.4 gC m^−2^ d^−1^; FLUXNET: −4.8 to 2.0 gC m^−2^ d^−1^), suggesting that spatial scale mismatches and meteorological forcing uncertainties might be the dominant sources of divergence. Previous studies have consistently shown that uncertainties associated with meteorological forcing can fundamentally alter modeled carbon fluxes and, in some regions, exceed the uncertainties arising from model structure itself (Bonan et al. 2019; Ryu et al. 2019; Seiler et al. 2021).

Third, fixed plant functional type parameterizations and incomplete representation of disturbance‐recovery dynamics limit the ability of models to capture NEE variability (Friend et al. 2007; Dietze et al. 2011; Keenan, Davidson, et al. 2013). For example, disturbance‐recovery chronosequences from flux towers documented annual forest NEE as high as ~+1270 g C m^−2^ year^−1^ after disturbance and as low as ~−1180 g C m^−2^ year^−1^ in recovering stands (age < 100 years) (Amiro et al. 2010). Such strong post‐disturbance carbon sources and regrowth carbon sinks are easily muted when forest age and recovery dynamics are weakly represented or overly averaged. Such incomplete process representation can lead to a ~50% underestimation of the northern forest carbon sink (O'Sullivan et al. 2024). A model‐data fusion study further showed that, even when long‐term flux records and multiple data greatly reduced parameter uncertainty, structural errors in process representation remained a dominant constraint on simulated NEE variability (Ricciuto et al. 2011).

For FLUXNET‐based ML approaches, model‐observation divergence also arises from two main sources. One is the limited representativeness of the flux‐tower training network, which reduces transferability across underrepresented climates, ecosystem types, and disturbance regimes (Nathaniel et al. 2023). The other is the reliance on meteorological and remote‐sensing predictors, meaning that important processes such as disturbance legacies, soil carbon turnover, management history, and recovery from extremes may only be indirectly or incompletely represented (Gaber et al. 2024). As a result, FLUXNET‐based ML products perform best in reproducing the observed NEE range, but some mismatch with FLUXNET still remains.

### 3.2 How Much Carbon Is Lost Through Respiration?

#### 3.2.1 Lessons From FLUXNET

**FIGURE 3:**
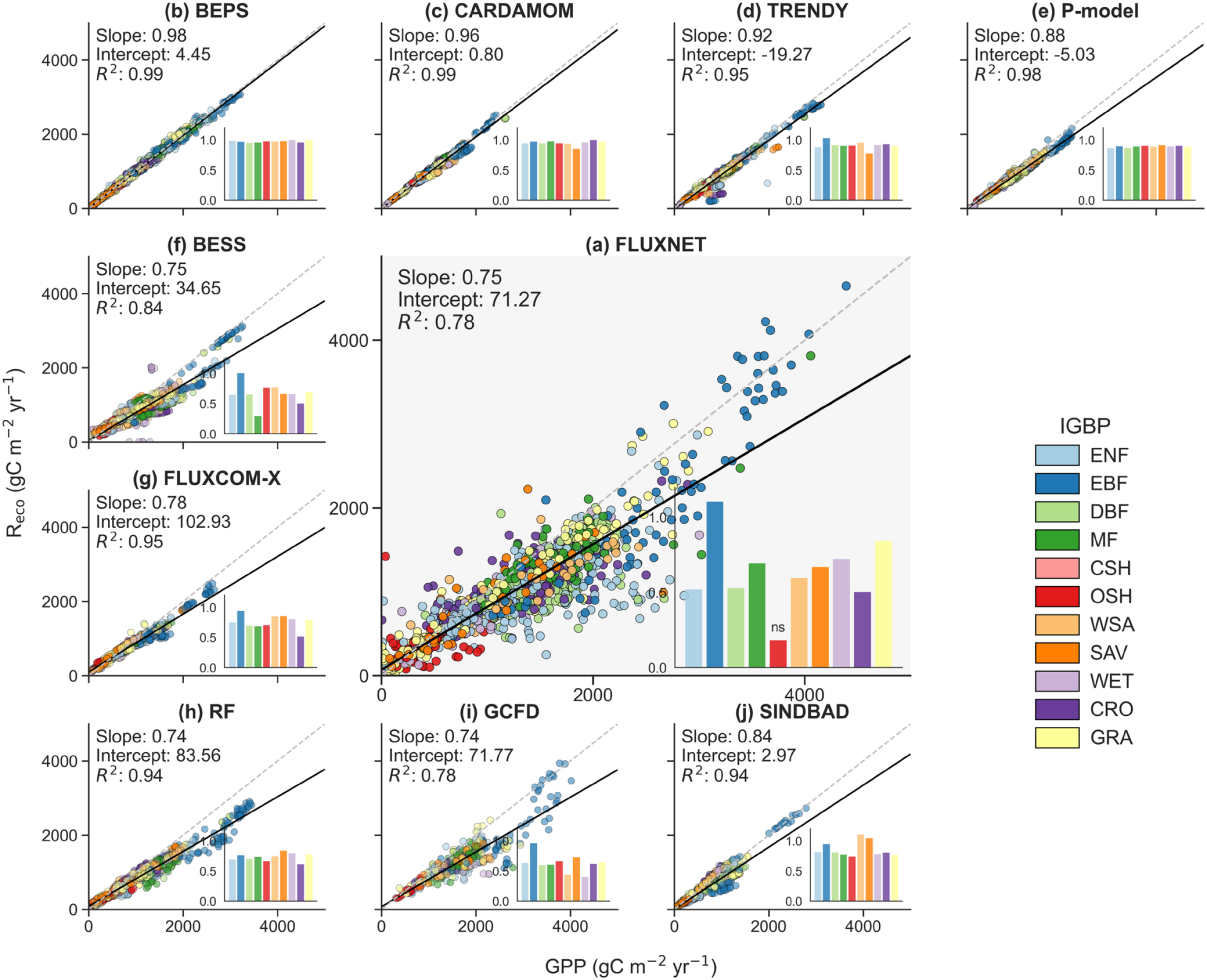
Relationships between Gross Primary Production (GPP) and Ecosystem Respiration (R_eco_), and regression slopes of R_eco_ vs. GPP across vegetation types for different models: (a) FLUXNET, (b) BEPS, (c) CARDAMOM, (d) TRENDY, (e) P‐model, (f) BESS, (g) FLUXCOM‐X, (h) RF, (i) GCFD, (j) SINDBAD. For all model products, annual GPP and R_eco_ were extracted for the same FLUXNET site‐years combinations as in panel (a), and bar graphs show the *R*_eco_‐GPP slope for each vegetation type. ‘ns’ indicates non‐significant regressions, while all others are significant (*p* < 0.05).

GPP and R_eco_ exhibit a tight spatiotemporal coupling (Janssens et al. 2001; Chen et al. 2015; Xiao et al. 2025). Their regression slope of R_eco_ on GPP (R_eco_‐GPP slope) could be relevant to carbon use efficiency, a “knob” that controls the conversion of GPP into NEE (Baldocchi and Penuelas 2019; Baldocchi 2020; Luo et al. 2025). FLUXNET measurements provided crucial empirical constraints on this relationship (Baldocchi 2008). Analysis of FLUXNET observations (Figure 3a) revealed that on an annual scale, approximately 75% of assimilated carbon is returned to the atmosphere through R_eco_, with this proportion varying among vegetation types (0.18–1.10). These different patterns likely reflect their distinct carbon allocation strategies and turnover rates (Nobel 1999; Malhi et al. 2009; Yang et al. 2021). Notably, slopes exceeding 1.0, indicating ecosystems functioning as net carbon sources, were predominantly associated with disturbance‐induced carbon losses. For example, logged tropical forests were reported to remain net carbon sources for a decade, as carbon losses from enhanced decomposition of deadwood and soil organic matter (Saleska et al. 2003; Huang and Asner 2010; Mills et al. 2023). In addition, the capacity of GPP to explain 78% of R_eco_ variations (*R* ^2^ = 0.78) quantified the degree of coupling between the two fluxes, though substantial variability remained unexplained. This unexplained variation likely suggested the influence of additional factors including temperature and soil moisture (Davidson and Janssens 2006; Bond‐Lamberty and Thomson 2010; Zhang et al. 2024), soil properties and nutrient (Litton et al. 2007; Zhang et al. 2019), stand age and disturbance history (Baldocchi 2008; Goulden et al. 2011; Chen et al. 2020). GPP and R_eco_ were derived from FLUXNET using the daytime partitioning method with variable u* threshold (DT_VUT_REF). Although different partitioning methods yield slightly different GPP and R_eco_ values, a comparison across four partitioning approaches (NT_VUT_REF, DT_VUT_REF, NT_CUT_REF, and DT_CUT_REF) confirmed that the R_eco_‐GPP relationship remains consistent (Figure S4). Understanding whether current models accurately capture variability of R_eco_‐GPP relationships is essential for evaluating the reliability of projected carbon cycle climate feedbacks.

#### 3.2.2 Model Evaluations Against FLUXNET

When evaluated using the same site‐years as in the FLUXNET analysis, different models exhibited distinct patterns in the R_eco_‐GPP relationship, including differences in both the R_eco_‐GPP slope and the degree of coupling (Figure 3b–i). In terms of the R_eco_‐GPP slope, process‐based models (BEPS, CARDAMOM, TRENDY, and P‐models) produced slope values close to 1 (0.88–0.98), with relatively small differences between different vegetation types. In contrast, BESS and FLUXNET‐based ML models (FLUXCOM‐X, RF and GCFD) generated slopes (0.74–0.78) consistent with FLUXNET observations and revealed clear biome differences. The hybrid model SINDBAD demonstrated a R_eco_‐GPP slope of 0.84, which fell between the higher slopes of the process‐based models and the lower slopes of the FLUXNET‐based ML models, while preserving substantial vegetation‐type variability. In terms of *R* ^2^, the process‐based models except BESS produced very high *R* ^2^ values (0.95–0.99), indicating too strong coupling between GPP and R_eco_. Among the FLUXNET‐based ML models, FLUXCOM‐X and RF also yielded high *R* ^2^ values (0.94–0.95), comparable to those of the process‐based models, whereas GCFD showed a lower *R* ^2^ of 0.78. The hybrid model SINDBAD had an *R* ^2^ of 0.94, indicating a degree of coupling between GPP and R_eco_ comparable to that of FLUXCOM‐X, RF and most process‐based models. When the annual R_eco_‐GPP relationship was further decomposed into spatial variability (Figure S5, multi‐year mean at each site) and temporal variability (Figure S6, site‐level standardized anomalies), the spatial analysis largely preserved the model differences already seen in Figure 3. By contrast, the temporal anomaly analysis showed that only RF and especially GCFD reproduced the FLUXNET relationship more closely, whereas most other models showed steeper slopes and over‐coupled R_eco_ to GPP in interannual variability.

#### 3.2.3 Sources of Model‐Observation Divergence

The differences in R_eco_‐GPP slopes between models are primarily determined by how each framework partitions GPP between respiration and carbon storage. Several process‐based models (Figure 3b–e) produced R_eco_‐GPP relationships close to the 1:1 line, with limited variation across vegetation types. As discussed in Section 3.1, most process‐based models simulated NEE as a narrow range of weak carbon sinks close to zero, implying small annual differences between GPP and R_eco_. In reality, R_eco_ continues year‐round while GPP ceases during dormant periods, creating a positive intercept in the R_eco_‐GPP relationship (Baldocchi and Penuelas 2019). This intercept reflects R_eco_ components not synchronized with contemporaneous GPP, including maintenance respiration supported by stored carbohydrates (Czimczik et al. 2006; Aubrey and Teskey 2018), decomposition of slowly cycling soil carbon pools (Luo et al. 2015; Wang et al. 2017; Pries et al. 2023), and legacy carbon from past disturbances (Amiro et al. 2010; Russell et al. 2014; Harmon et al. 2020). These process‐based models exhibited smaller intercepts than those inferred from FLUXNET observations, further shifting the fitted R_eco_‐GPP relationship toward the 1:1 line. FLUXNET‐based ML models learn empirical relationships from meteorological and remote‐sensing predictors to site‐level GPP and R_eco_. Because their predictor variables include plant functional type and leaf area index (LAI)‐related variables that capture differences among vegetation types (Zeng et al. 2020; Shangguan et al. 2023; Nelson et al. 2024), they reproduce R_eco_‐GPP slopes more consistent with the FLUXNET observations. In SINDBAD, the coupled carbon‐water processes in the model framework are governed by a set of globally constant parameters, whereas CARDAMOM performs Bayesian optimization of parameters for each grid cell (Lee et al. 2025). Although grid‐cell level optimization allows parameters to adapt to local ecosystem characteristics, it risks overfitting to site‐specific conditions and can result in an overly tight coupling between R_eco_ and GPP. In contrast, globally constant parameters in SINDBAD, combined with a soil‐carbon‐related background respiration term that is partially decoupled from current photosynthetic inputs, produce a R_eco_‐GPP slope (0.84) that more closely approaches the FLUXNET‐observed relationship. Therefore, R_eco_ increases more slowly than GPP on the annual scale, and the R_eco_‐GPP relationship deviates more from the 1:1 line than in CARDAMOM and the other process‐based models.

Variations in *R* ^2^ among models mainly depend on how respiration is structurally linked to assimilated carbon in each framework. Except for BESS and GCFD, annual R_eco_ and GPP are more tightly coupled than in the FLUXNET benchmark. In most process‐based models, autotrophic respiration is parameterized using fixed plant functional type constants, yielding approximately stable NPP/GPP ratios (Del et al. 2007; Randerson et al. 2025), while heterotrophic respiration is represented as first‐order decay of a limited number of soil and litter carbon pools, driven by simple environmental scalars of temperature and soil moisture (Sierra et al. 2012; Guenet et al. 2016; Luo et al. 2016). This formulation neglects microbial controls and disturbance legacies, causing heterotrophic respiration to co‐vary tightly with GPP (Schimel and Schaeffer 2012; Todd‐Brown et al. 2013; Guenet et al. 2016). For FLUXNET‐based ML models, the contrast in GPP‐R_eco_ coupling mainly stems from how the GPP, R_eco_ and NEE are preprocessed and constrained in the upscaling frameworks. In the RF model, daily in situ fluxes were aggregated to 10‐day means and GPP was recalculated from R_eco_ and NEE after screening out poorly closed records (Zeng et al. 2020). FLUXCOM‐X upscales GPP and NEE, and R_eco_ is diagnosed as the difference between these two fluxes (Nelson et al. 2024). By contrast, in the GCFD model, GPP, R_eco_ and NEE are modelled as separate targets, which allows R_eco_ to vary more independently from GPP and results in weaker GPP‐R_eco_ coupling than in the other two FLUXNET‐based ML models (Shangguan et al. 2023).

### 3.3 How Much Water Is Used to Fix Carbon?

#### 3.3.1 Lessons From FLUXNET

**FIGURE 4:**
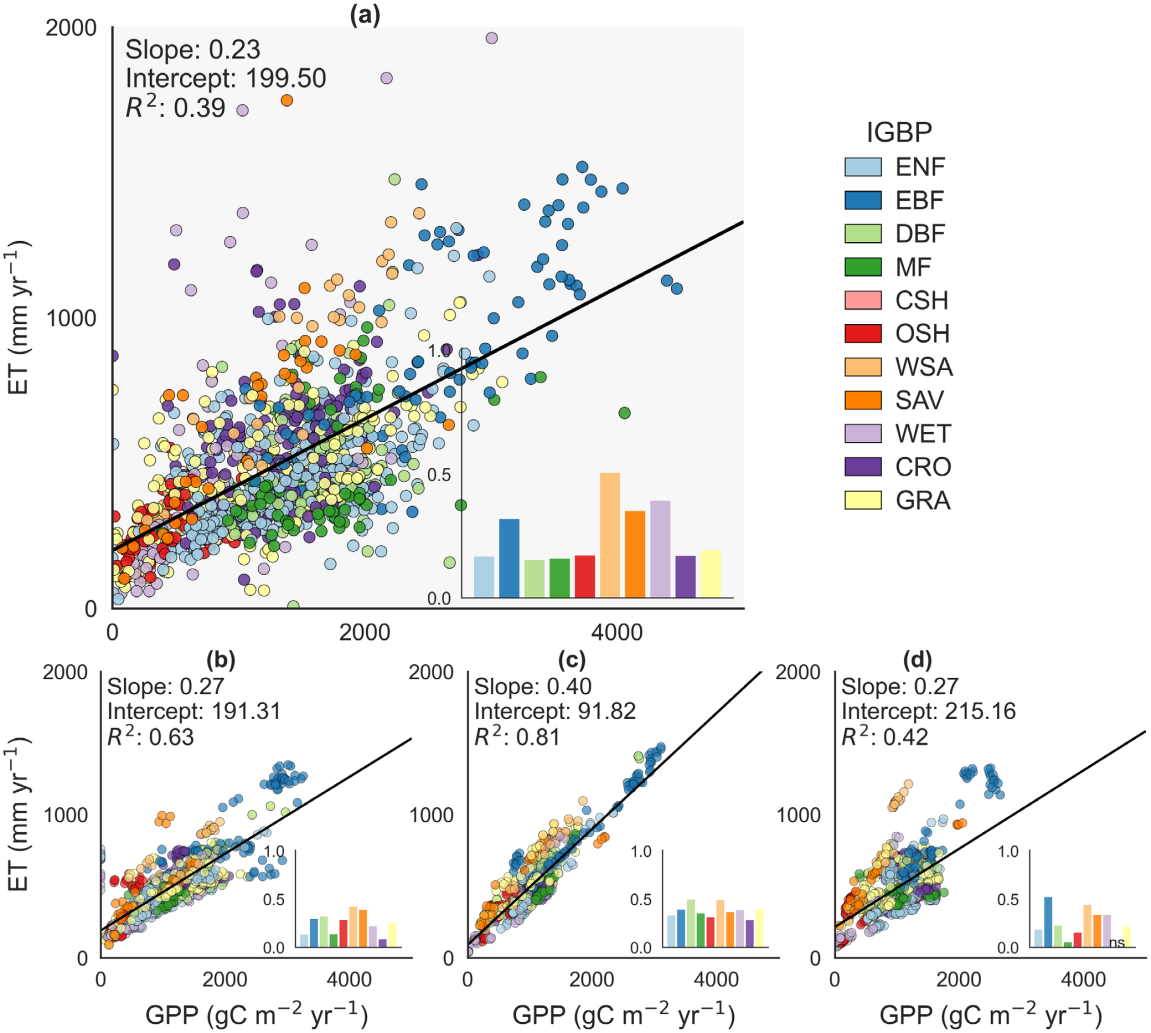
Relationships between GPP and evapotranspiration (ET), and regression slopes of ET vs. GPP across vegetation types for different models: (a) FLUXNET, (b) BESS, (c) TRENDY, and (d) FLUXCOM‐X. For all model products, annual GPP and ET were extracted for the same FLUXNET site‐year combinations as in panel (a), and bar graphs show the ET‐GPP slope for each vegetation type. ‘ns’ indicates non‐significant regressions, while all others are significant (*p* < 0.05).

FLUXNET observations provide critical benchmarks for understanding how water availability constrains the land carbon sink through carbon‐water coupling. Long‐term eddy‐covariance syntheses have demonstrated a close correlation between ecosystem carbon uptake and ET (Law et al. 2002; Tang, Li, et al. 2014). Water availability and evaporative demand emerge as key regulators of land carbon sink variability, particularly in semi‐arid and drought‐prone regions that play a dominant role in variations of the global land carbon sink (Poulter et al. 2014; Ahlström et al. 2015; Barnes et al. 2021). The FLUXNET observations indicate an ET‐GPP regression slope of about 0.23 kg H_2_O gC^−1^, meaning that an increase of 1000 gC m^−2^ year^−1^ in GPP is associated with roughly 230 mm year^−1^ of ET returned to the atmosphere (Figure 4a). The intercept of ~199 mm year^−1^ suggests substantial water loss from soil evaporation and canopy interception, even when GPP approaches zero. Moreover, the *R* ^2^ of 0.39 between FLUXNET GPP and ET suggests the complexity of carbon‐water cycling at the ecosystem scale. For example, annual GPP and ET are strongly coupled in semi‐arid and arid regions, but in humid or energy‐limited regions, they are only weakly related (Zhang et al. 2016). Furthermore, differences in climate, vegetation structure and nutrient availability among sites lead to pronounced variation in GPP‐ET relationships (Tang, Luyssaert, et al. 2014; Guerrieri et al. 2016; Wang et al. 2021).

#### 3.3.2 Model Evaluations Against FLUXNET

Against this FLUXNET benchmark, models show different water costs for sustaining a given carbon uptake. When evaluated over the same FLUXNET site‐years (Figure 4b–c), BESS and FLUXCOM‐X reproduced ET‐GPP slopes close to the observational estimate (~0.27 kg H_2_O gC^−1^ versus 0.23 kg H_2_O gC^−1^) and intercepts in a similar range (~191–215 mm year^−1^ versus ~199 mm year^−1^), indicating broadly consistent water use per unit carbon uptake and comparable contributions from soil evaporation and canopy interception. In contrast, the TRENDY ensemble exhibits a much steeper ET‐GPP slope of about 0.40 kg H_2_O gC^−1^ and a lower intercept of ~92 mm year^−1^, implying that the TRENDY models require substantially more ET to achieve the same increase in GPP and that little ET occurs in the absence of photosynthesis. In addition, the notably higher R^2^ values between GPP and ET in both TRENDY (0.81) and BESS (0.63) compared to FLUXNET observations (0.39) suggest that process‐based models tend to represent a tighter carbon‐water coupling than that inferred from FLUXNET observations. From the perspective of carbon sink estimation, these differences suggest that TRENDY tend to represent a carbon uptake that was more directly constrained by water availability than indicated by the FLUXNET benchmark. A similar partitioning of the annual ET‐GPP relationship into cross‐site means and site‐level standardized anomalies provided further insight into model behavior (Figures S7 and S8). The spatial analysis broadly preserved the same inter‐model differences as in the combined annual site‐year relationship, whereas the anomaly‐based analysis indicated systematically stronger ET‐GPP coupling in models than in FLUXNET, suggesting that interannual carbon‐water variability is still not well captured.

#### 3.3.3 Sources of Model‐Observation Divergence

The divergence between modeled and observed GPP‐ET relationships likely indicates inadequate representation of rising CO_2_ effects on carbon‐water coupling. The coupling strength between GPP and ET in the TRENDY models is higher than that derived from FLUXNET observations. Recent analyses of northern ecosystems showed that the observed correlation between GPP and ET weakened substantially over the past two decades (Zhao, Luo, et al. 2025). Many models in the TRENDY ensemble do not reproduce this decoupling and instead maintain a tight GPP‐ET coupling (Zhao, Shi, et al. 2025). This weakening was mainly attributed to rising atmospheric CO_2_, which increases GPP while reducing stomatal conductance and transpiration, thereby decoupling carbon and water fluxes (Keenan, Hollinger, et al. 2013; Li, Xiao, et al. 2023). In other words, TRENDY represents a higher water cost and lower ecosystem water use efficiency than suggested by the FLUXNET benchmark. This interpretation was consistent with previous evidence that many land‐surface models underrepresent CO_2_‐driven increases in water use efficiency and over‐attribute interannual variability of GPP to soil‐moisture fluctuations (Humphrey et al. 2021). In contrast, FLUXCOM‐X predicts GPP and ET as separate variables in a data‐driven upscaling framework, rather than enforcing a tight coupling between them. This distinction is also consistent with recent hybrid modeling work in which the machine‐learning component provides more flexible model formulations, allowing carbon‐water coupling to be less rigid and enabling a wider range of coupling strengths across environmental conditions (Fang and Gentine 2024). At the global scale, rising CO_2_, increasing atmospheric and soil dryness and more frequent climate extremes are reshaping the links between photosynthesis, transpiration and NEE, highlighting the need to confront models with joint constraints on both carbon and water fluxes (Gentine et al. 2019).

### 3.4 Influence of Forest Age, Forest Management Types, and Nutrient Limitations on GPP‐R_eco_ Relationship

#### 3.4.1 Forest Age

##### 3.4.1.1 Background

The capacity of forests to act as carbon sinks changes systematically with stand age, as the balance between GPP and R_eco_ shifts over succession (Odum 1969; Curtis and Gough 2018). Several studies have shown that NEP typically peaks in middle‐aged stands and declines as forests aged, even though some old forests remained weak but persistent carbon sinks (Luyssaert et al. 2008; Tang, Luyssaert, et al. 2014; Gough et al. 2016). At regional to global scales, global forest inventory and modelling studies have likewise revealed that forests in the regrowth phase, typically a few decades to about a century after disturbance, occupy only part of the forest area but provide a large share of the contemporary forest carbon sink (Pan et al. 2011; Pugh et al. 2019). Thus, newly established and regrowing forests play a crucial role in offsetting the weakening of carbon sink associated with forest aging and deforestation (Leng et al. 2024; Peng et al. 2025; Besnard et al. 2025).

##### 3.4.1.2 Lessons From FLUXNET

**FIGURE 5:**
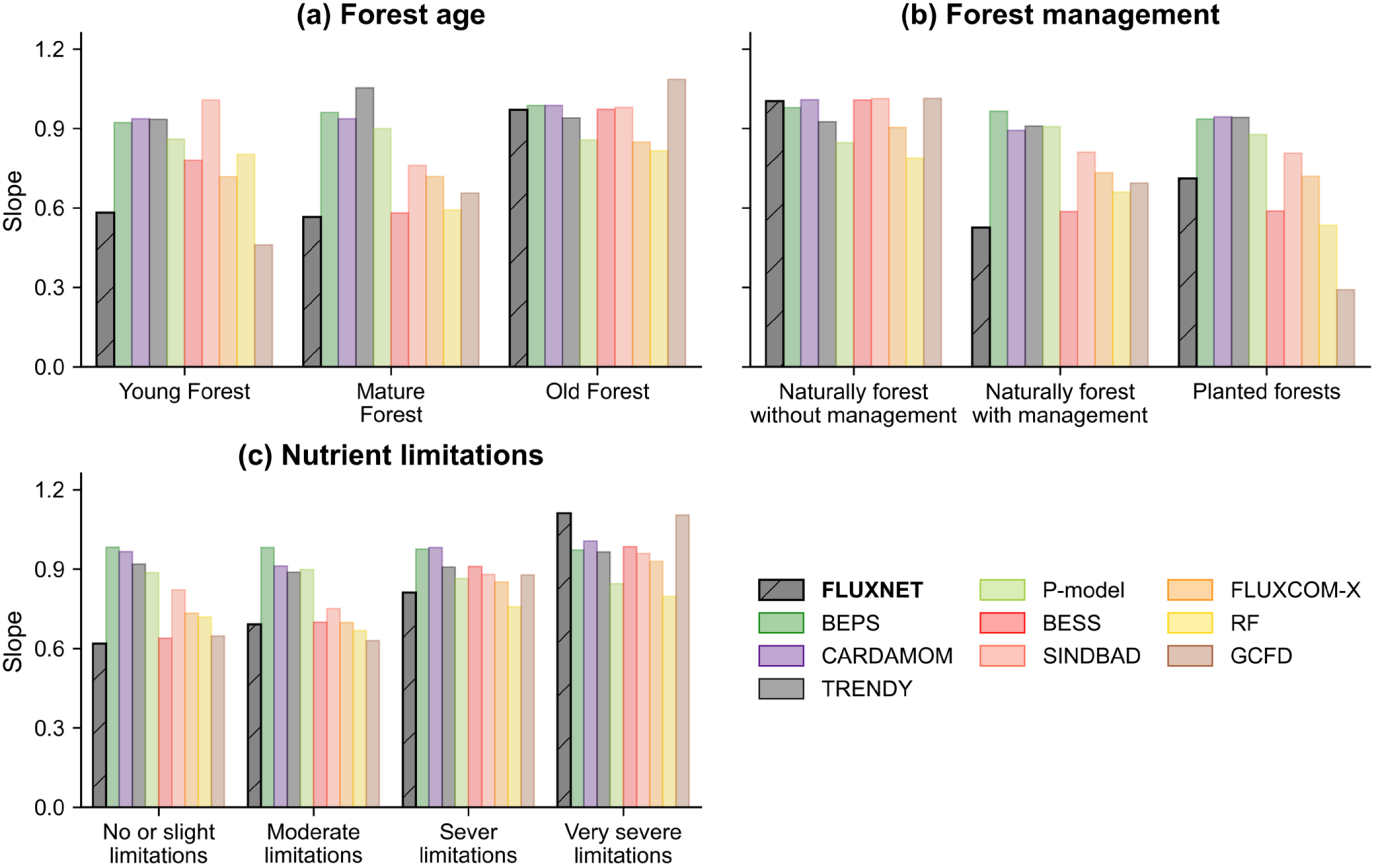
Regression Slopes of R_eco_ vs. GPP for various (a) Forest age classes (Young Forest: ≤ 40 year; Mature Forest: 40–100 year; Old Forest: > 100 year, following Ameray et al. (2023)), (b) Forest management types, and (c) Nutrient limitations across different models. Data sources include: Global forest age (Besnard et al. 2021); Forest management type (Lesiv et al. 2022); Nutrient limitations (Harmonized World Soil Database v 1.2, Fischer et al. 2008). All regressions were statistically significant (*p* < 0.05). For all model products, annual GPP and R_eco_ were extracted for the same FLUXNET site‐year combinations.

FLUXNET data provide a quantitative view of how forest age affected the balance between GPP and R_eco_. Numerous studies have utilized eddy covariance observation networks to investigate the impact of forest age on carbon sinks (Litvak et al. 2003; Grant et al. 2007; Amiro et al. 2010). Meta‐analyses of eddy covariance networks further have demonstrated that this age pattern of carbon sink is primarily driven by changes in GPP, with GPP declining faster than R_eco_ with increasing forest age (Tang, Luyssaert, et al. 2014). Based on FLUXNET data, we observed a similar pattern: young and mature forests exhibited lower slopes (~0.6), indicating that a larger proportion of assimilated carbon was retained, whereas old forests had slopes approaching 1, reflecting increased respiration that nearly offset carbon uptake (Figure 5a). These results reinforce that the contemporary global forest sink was heavily supported by regrowing and middle‐aged stands.

##### 3.4.1.3 Model Evaluations Against FLUXNET

However, most global models do not fully capture the age gradient in the GPP‐R_eco_ relationship revealed by FLUXNET, indicating that forest age and successional processes are still represented too coarsely. FLUXNET‐based ML models and BESS captured the qualitative pattern of rising slopes from young to old forests but tend to overestimate slopes for young and mature stands (Figure 5a). Other process‐based models generally produced high and relatively uniform slopes across all age classes, implying weak sensitivity of carbon sink estimates to forest age. This behavior was consistent with broader assessments that current DGVMs and land‐surface models under‐represent the effects of disturbance history and age structure on carbon fluxes (Pugh et al. 2019; O'Sullivan et al. 2024). Although some models might partly capture stand development through temporal changes in LAI and canopy structure (Peng et al. 2025), they still do not represent stand demography explicitly, so age‐related shifts in allocation, mortality, and decomposition are strongly damped. In contrast, models that explicitly include forest age, for example, by dividing stands into age classes or by using forest‐age maps to prescribe stand age, showed that changes in forest age‐class distribution alone produce a substantial but transient carbon sink and substantially change the simulated spatial pattern of forest carbon uptake (Pugh et al. 2019; Leng et al. 2024; Zhang et al. 2025). Therefore, models need to represent forest age and stand development explicitly, and to use forest‐age maps together with eddy‐covariance site constraints to predict how forest carbon sinks will evolve as newly established stands mature.

#### 3.4.2 Forest Management

##### 3.4.2.1 Background

Forest management alters the balance between carbon uptake and respiration by thinning, harvesting, and implementing silvicultural and restoration strategies (Ontl et al. 2020; Yu et al. 2021; Pan et al. 2024). Comparing managed and unmanaged forests shows that management often reduces total ecosystem carbon stocks, especially in soils, but can increase tree growth and aboveground biomass (Noormets et al. 2015; Ameray et al. 2021), and managed forests also tend to show higher biomass production efficiency, consistent with nutrient‐enabled allocation towards wood growth (Campioli et al. 2015). At regional scales in China, analyses and simulations have further suggested that future forest sinks are highly sensitive to management intensity and forest expansion, and that sink strength can weaken as stands age even under similar climate forcing (Tong et al. 2020; Yu et al. 2021). Together, these studies indicate that forest management is a key driver of where and when forests act as strong and persistent carbon sinks.

##### 3.4.2.2 Lessons From FLUXNET

Eddy‐covariance measurements have revealed how management practices alter the R_eco_‐GPP relationship at the stand scale. For example, eddy‐covariance studies at thinned or partially harvested forests showed that strong interventions often turn forests into temporary carbon sources, followed by a recovery period during which carbon sink gradually increases as forest stands regrow (Dore et al. 2012; Saunders et al. 2012; Lindroth et al. 2018). In our FLUXNET analysis (Figure 5b), naturally forests without management, which often include later successional or older forest, showed the steepest R_eco_‐GPP slopes, indicating higher respiration relative to GPP and therefore weaker annual carbon sinks. By contrast, managed naturally regenerated forests and planted forests had lower slopes and acted as stronger sinks. Overall, these patterns suggest that management increases carbon sink strength by maintaining stands in fast‐growing stages or favoring high‐growth species, thereby reducing the respiratory cost per unit GPP (Campioli et al. 2015; Noormets et al. 2015).

##### 3.4.2.3 Model Evaluations Against FLUXNET

However, most models fail to capture forest management effects on the carbon sink. The FLUXNET‐based ML model and BESS captured the broad contrast between naturally unmanaged forests and other forest types, but they remain less sensitive to the distinction between planted forests and naturally managed forests. By contrast, most process‐based models maintain similarly high and nearly invariant R_eco_‐GPP slopes across management classes, showing that management barely changes the balance between R_eco_ and GPP, likely because key pathways such as explicit management operations and stand age are only weakly represented. Many DGVMs in the TRENDY protocol include wood harvest and forest degradation (Friedlingstein et al. 2023), but explicit forest age structure is not consistently represented. Consequently, harvest often functions as carbon removal rather than triggering realistic regrowth dynamics. This limitation matches broader DGVM assessments showing that models based on grid‐cell average vegetation states struggle to represent disturbance recovery unless they track plant size or age structure (Argles et al. 2022). Consistent with this, studies that explicitly represent forest age dynamics have shown that changes in age structure alone generate a substantial but temporary carbon sink in regrowing forests and alter the inferred contribution of regrowth to contemporary net uptake (Haverd et al. 2018; Yue et al. 2018; O'Sullivan et al. 2022). Overall, explicitly accounting for demographic shifts in regrowth forests reveals a carbon sink mechanistically distinct from environmental forcing, confirming that detailed stand age and size are essential for correctly simulating the carbon balance of managed ecosystems.

#### 3.4.3 Nutrient Limitations

##### 3.4.3.1 Background

Nutrient limitations could affect carbon supply and growth differently, thereby regulating carbon sequestration capacity of terrestrial ecosystems (Fatichi et al. 2014; Wieder et al. 2015; Terrer et al. 2019). Higher nutrient availability tends to strengthen net carbon uptake by reducing the fraction of gross production lost through R_eco_, partly via shifts in allocation toward longer‐lived tissues and slower carbon turnover (Vicca et al. 2012; Fernández‐Martínez et al. 2014). Conversely, nutrient limitation imposes negative feedback on photosynthesis that can even offset CO_2_‐fertilization gains by limiting tissue growth, shifting allocation and turnover (Fatichi et al. 2014; Fleischer and Terrer 2022; Ruehr et al. 2023).

##### 3.4.3.2 Lessons From FLUXNET

In our FLUXNET analysis, the slope of the GPP‐ R_eco_ regression increased from sites with no/slight nutrient limitations to those with very severe limitations (Figure 5c), indicating that respiration accounts for a growing fraction of gross production under stronger nutrient constraints. All vegetation types were included when assessing the influence of nutrient limitations on the R_eco_‐GPP relationship. This pattern agrees with Fernández‐Martínez et al. (2014), which showed that nutrient availability dominates forest NEE, and stronger nutrient limitation increases the fraction of GPP lost to respiration. It also aligns with evidence that more fertile systems tend to convert photosynthates into biomass more efficiently (Vicca et al. 2012) and that nitrogen inputs can suppress soil carbon losses in many cases, via reduced decomposition rates (Janssens et al. 2010).

##### 3.4.3.3 Model Evaluations Against FLUXNET

Most process‐based models showed small variations in R_eco_‐GPP slopes across nutrient‐limitation classes (Figure 5c). In contrast, FLUXNET‐based ML models better reproduced the increasing slope pattern from nutrient‐sufficient to nutrient‐limited sites, despite not explicitly incorporating nutrient data as an input variable. This pattern indicates that the fraction of GPP lost to respiration is too large under nutrient‐sufficient conditions and does not increase enough under nutrient stress. In these models, nutrient effects are either omitted or represented primarily as simple limits on GPP, while R_eco_ and decomposition remain governed largely by climate scalars and prescribed turnover rates. These models can match the global land carbon sink, yet still disagree on whether it mainly comes from vegetation growth or soil carbon change, and even carbon‐nitrogen coupled models often fail to reproduce observed nitrogen cycling (O'Sullivan et al. 2022; Kou‐Giesbrecht et al. 2023; Vallicrosa et al. 2025). These issues matter because nutrient limitation can substantially alter future sink projections and remaining carbon budgets, especially when phosphorus limitation is considered (Wieder et al. 2015; de Sisto and MacDougall 2024). Recent work has also shown that even carbon‐phosphorus models could overestimate CO_2_‐enrichment responses if key nutrient‐microbe constraints are not represented realistically (Jiang et al. 2024). In practice, models should reproduce how nutrient limitation regulates the R_eco_‐GPP balance, which likely requires linking nutrient supply to growth, allocation and turnover, and soil decomposition rather than adjusting photosynthesis alone (Fatichi et al. 2014).

## 4 Challenges of FLUXNET for Estimating the Global Land Carbon Sink

**FIGURE 6:**
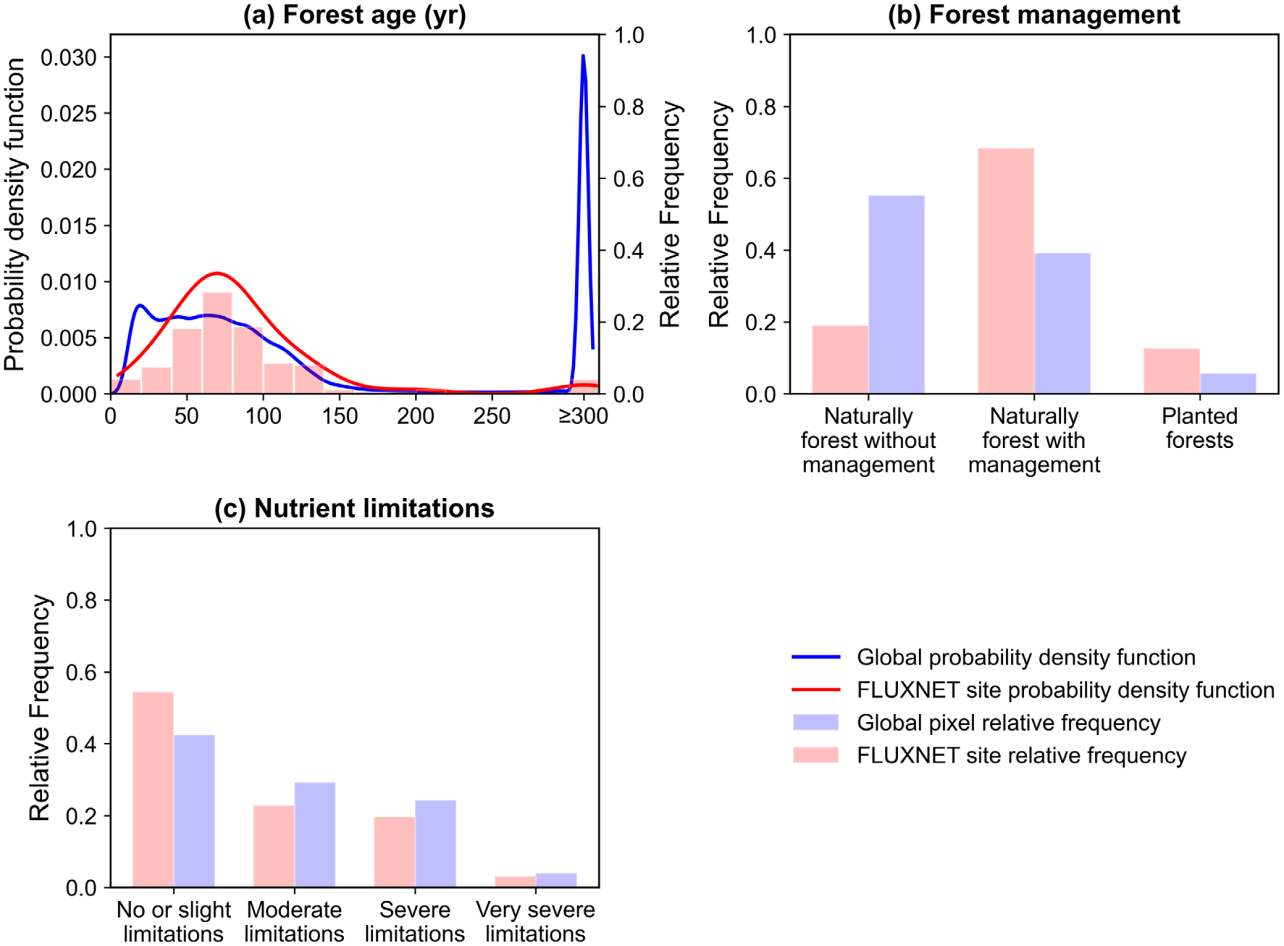
Distributions of forest age, management type, and nutrient limitation in global gridded data and at FLUXNET site locations. Comparisons are shown between global gridded conditions (all land pixels; blue) and the subset represented by FLUXNET site locations (pink). (a) Forest age distribution. Bars indicate relative frequency, and lines indicate the probability density function (blue, global; red, FLUXNET sites). The last bin represents forest ages ≥ 300 year. (b) Forest management composition, shown as relative frequency across naturally unmanaged forests, naturally managed forests, and planted forests. (c) Nutrient limitation classes, shown as relative frequency from no or slight limitation to very severe limitation.

Spatial representativeness constrains how far FLUXNET observations can be extrapolated to quantify the global land carbon sink (Kumar et al. 2016). Using the three factors affecting the R_eco_‐GPP slope from Section 3.4 as an example, FLUXNET sampling diverges from global conditions in forest age and management, while nutrient limitation exhibits a closer match with global distributions (Figure 6). These three factors are used here only as illustrative examples rather than an exhaustive set of representativeness dimensions. Similar limitations also apply to vegetation types and broader ecosystem functional properties, as flux observations remain uneven across land‐cover classes (see Figure 2 in Gaber et al. 2024), and additional challenges in tropical dryland and cold‐region ecosystems (Biederman et al. 2017; Mavrovic et al. 2023; Ryu 2024). FLUXNET site‐years skew toward mature stands, under‐sampling both very old forests (> 250 year) and very young forests (< 30 year)—the latter often associated with disturbance legacies and substantial CO_2_ sources (Luyssaert et al. 2008). FLUXNET also over‐represents managed, naturally regenerated forests while under‐represents unmanaged natural forests common in primary‐forest regions. As a result, FLUXNET‐based models might capture the right reasons within well‐sampled regions yet still fail to deliver the right answer for global carbon sink estimation due to biases in poorly sampled forest age classes and management regimes (Papale et al. 2015). These sampling biases directly affect FLUXNET‐based ML model performance across the factors discussed in Section 3.4. Young forests and planted forests are underrepresented in the training data, likely contributing to poorer FLUXNET‐based ML model performance for these categories. FLUXCOM‐X, which trains on hourly data rather than daily aggregates, benefits from a larger number of training samples, thereby reducing errors associated with limited site‐year coverage. To address these limitations, future efforts should prioritize expanding FLUXNET coverage and incorporating forest age, management status, and nutrient limitation as explicit predictor variables in FLUXNET‐based ML models.

Temporal representativeness further constrains inference across decades and under emerging extremes. Chu et al. (2017) have shown that finite measurement periods are uneven across sites and may not capture the full range of climatological and biological variability needed for robust temporal extrapolation, so extensions to earlier decades such as the 1980s require caution. This limitation matters because extremes often dominate interannual sink anomalies (Reichstein et al. 2013; Zscheischler et al. 2014; Ahlström et al. 2015). For example, the 2023 extreme heat events caused a global carbon loss of 1.73 Gt C year^−1^ (Ke et al. 2024), and the carbon sink decline was dominated by reduced photosynthesis in tropical regions (Du et al. 2025). The underlying mechanism involves an asymmetric response between GPP and R_eco_, where GPP declines more severely than R_eco_, disrupting the normal carbon balance (von Buttlar et al. 2018; Yu et al. 2022; Kannenberg et al. 2024). This challenge is amplified by the fact that extreme events often trigger threshold‐like ecosystem responses, for example when drought or heat stress exceeds physiological tolerances and causes abrupt changes in photosynthesis, respiration, or mortality risk. Such nonlinear feedbacks are difficult to represent in process‐based models and remain poorly learned by ML models when such events are rare in the training record. Therefore, even FLUXNET‐based models that appear to have the right reasons within the observed period might fail to provide the right answer for long‐term carbon sink estimation if extremes and recovery dynamics are under‐represented in the training data.

Flux measurement and processing uncertainties also limit how strongly tower data can constrain global carbon sink estimates. Although FLUXNET2015 applies standardized quality control and harmonization across sites (Pastorello et al. 2020), annual carbon budgets still depend on choices in turbulence filtering, gap filling, and flux partitioning, each introducing uncertainties that propagate into derived GPP and R_eco_ (Papale et al. 2006; Lasslop et al. 2010; Xiao et al. 2025). Additionally, commonly used partitioning approaches have been found to underestimate GPP and R_eco_ during rain pulse events in drylands, which implies that event‐scale dynamics can be systematically smoothed in derived component fluxes without bias correction (Nguyen et al. 2025). These uncertainties can steer model parameters toward compensating errors that reproduce processed tower products rather than robust process representations valid across event types and climates. To better account for these uncertainties, it is preferable to compare ensembles of alternative processing pathways, including multiple u* filtering thresholds, gap‐filling approaches, and flux partitioning methods, rather than relying on a single processed product (Moffat et al. 2007; Lucas‐Moffat et al. 2022; Vekuri et al. 2025). Incorporating emerging partitioning approaches into such ensembles may be particularly valuable, as these methods can better represent complex ecosystem dynamics that are often missed by conventional schemes (Tramontana et al. 2020; Nguyen et al. 2025).

Additional observational constraints can complement FLUXNET data and provide further process constraints for process‐based models. Solar‐induced chlorophyll fluorescence (SIF), carbonyl sulfide (COS or OCS), and carbon isotope discrimination (Δ^13^C) provide complementary windows into photosynthesis rather than redundant information (Chen et al. 2017; Norton et al. 2018; Kooijmans et al. 2021). Measurements of OCS and SIF, for example, provide complementary constraints on different aspects of photosynthetic activity: OCS uptake differs fundamentally from CO_2_ exchange because it is largely a one‐way flux through stomata followed by rapid hydrolysis inside leaves, and therefore provides a distinct constraint on stomatal uptake processes (Keeling and Graven 2021; Whelan et al. 2020). SIF, in contrast, contains information related to photosynthetic functioning, although the observed signal is also strongly modulated by canopy structure (Dechant et al. 2020; Sun et al. 2017). By constraining different steps of the same process, these tracers can help improve the mechanistic representation and parameterization of models, thereby leading to more reliable estimates of the land carbon sink.

Nevertheless, even if spatial and temporal representativeness improve and processing uncertainties are better characterized, these advances may enhance model evaluation and the training of data‐driven models, while their contribution to mechanistic understanding is likely to remain limited. Covariation among climate, soil, vegetation, and management factors cannot be fully disentangled from observational data alone. Coordinated large‐scale experimental manipulations, including free‐air CO_2_ enrichment and distributed experimental networks such as Nutrient Network, provide opportunities to isolate causal drivers and strengthen process‐level understanding (Borer et al. 2014; Fraser et al. 2013; Raoult et al. 2024). Expanding such experimental frameworks across multiple flux tower sites, although costly, could provide a more comprehensive pathway to obtaining the right answer for the right reasons by directly testing the mechanisms underlying carbon sink responses to global change.

## 5 Conclusion

This review reveals a fundamental challenge in land carbon sink estimation: models achieving reasonable global annual NEE estimates often misrepresent underlying processes, while those capturing site‐scale mechanisms might produce unrealistic global estimates. The order‐of‐magnitude spread in global NEE estimates arises not only from technical uncertainty but also from contrasting representations of carbon‐related couplings across ecosystems. Several process‐based and top‐down products achieve plausible global mean NEE while producing overly narrow site‐level NEE distributions, and many models do not reproduce FLUXNET‐informed insights into R_eco_‐GPP slopes, GPP‐ET coupling, and their sensitivities to forest age, management, and nutrient limitations. FLUXNET‐based ML models reproduced these observational relationships more closely but remain constrained by training‐data representativeness, as FLUXNET sampling emphasizes mature, managed temperate forests while undersampling tropical, old‐growth, and disturbed systems. Moving forward requires expanded observational coverage in underrepresented regions, with the forthcoming FLUXNET2025 release expected to partially address these sampling biases. Process representation needs improvement through explicit incorporation of age‐dependent traits, management effects, and nutrient limitation, while the increasing frequency of extreme events demands approaches capable of capturing nonlinear responses and legacy effects. Integration of different methodological approaches, as demonstrated by hybrid frameworks such as SINDBAD and ATM_EC, offers promising pathways for reducing uncertainties because it combines the large‐scale budget constraints of top‐down approaches with the finer ecological information and temporal variability captured by bottom‐up observations and process‐based representations (Upton et al. 2024; Lee et al. 2025; Pandey et al. 2025). Meanwhile, hybrid approaches that combine process‐based models with machine learning offer a complementary pathway by improving the representation of poorly constrained processes while retaining a mechanistic basis (Fang and Gentine 2024; Worden et al. 2025). Additional observational constraints—including OCS, SIF, and Δ^13^C—could further complement model evaluation and parameter optimization. Through such systematic integration, we can progress toward achieving both the right answer and the right reasons for global land carbon sink estimation.

## Author Contributions

**Helin Zhang:** conceptualization, data curation, formal analysis, investigation, methodology, visualization, writing – original draft, writing – review and editing. **Youngryel Ryu:** funding acquisition, investigation, methodology, project administration, resources, supervision, writing – review and editing. **Sungchan Jeong:** conceptualization, investigation, writing – review and editing.

## Funding

This work was supported by the National Research Foundation of Korea, RS‐2024‐00348585.

## Conflicts of Interest

The authors declare no conflicts of interest.

## Supporting information


**Figure S1:** Spatial patterns of mean annual net ecosystem exchange (NEE) across 16 global.
**Figure S2:** Spatial patterns of net ecosystem exchange (NEE) trends from representative.
**Figure S3:** Overview of FLUXNET sites used in this study. (a) Spatial distribution of sites overlaid on global climate zones, with symbols indicating IGBP.
**Figure S4:** Comparison of R_eco_‐GPP relationships derived from four flux partitioning methods.
**Figure S5:** Cross‐site GPP‐R_eco_ relationships based on multi‐year mean values at each.
**Figure S6:** Interannual GPP‐R_eco_ relationships based on year‐to‐year anomalies at each.
**Figure S7:** Cross‐site GPP‐ET relationships based on multi‐year mean values at each.
**Figure S8:** Interannual GPP‐ET relationships based on year‐to‐year anomalies at each.

## Data Availability

FLUXNET data are available at https://fluxnet.org/. TopRMB2‐down model products include OCORMB2‐2 v10 MIP (https://gml.noaa.gov/ccgg/OCO2_v10mip/), CarboScope inversion (https://www.bgcRMB2‐jena.mpg.de/CarboScope/?ID=sEXTocNEET_v2022; DOI: 10.17871/CarboScopeRMB2‐sEXTocNEET_v2022), and CarbonTracker CT2022 (https://doi.org/10.25925/z1gjRMB2‐3254). ProcessRMB2‐based model outputs can be accessed as follows: BEPS from the National Ecosystem Science Data Center, China (http://www.nesdc.org.cn; DOI: 10.12199/nesdc.ecodb.2016YFA0600200.02.003), TRENDY and CARDAMOM (https://mdosullivan.github.io/GCB/), and BESS (https://www.environment.snu.ac.kr/bessv2). Hybrid model products include SINDBAD (https://doi.org/10.5281/zenodo.11388020) and ATM_EC (https://doi.org/10.5281/zenodo.10454297). FLUXNETRMB2‐based ML products are accessible at: FLUXCOMRMB2‐X (https://doi.org/10.18160/5NZGRMB2‐JMJE), FLUXCOM_RS and FLUXCOM_RS+METEO (https://www.bgcRMB2‐jena.mpg.de/geodb/projects/Data.php), RF (https://doi.org/10.17595/20200227.001), and GCFD (https://doi.org/10.11888/Terre.tpdc.300009). SVR (Kondo et al. 2015) and PRMB2‐model (Cai et al. 2025) data are available upon request. Fire emissions data were obtained from Global Fire Emissions Database version 4.1 (https://doi.org/10.3334/ORNLDAAC/1293). Global forest age data were derived from https://doi.org/10.17871/ForestAgeBGI.2021. Forest management type data were sourced from https://doi.org/10.5281/zenodo.5849150. Nutrient limitation data were extracted from the Harmonized World Soil Database v1.2 (https://www.fao.org/soilsRMB2‐portal/dataRMB2‐hub/soilRMB2‐mapsRMB2‐andRMB2‐databases/harmonizedRMB2‐worldRMB2‐soilRMB2‐databaseRMB2‐v12/en/).
