# Supplementary material for: Towards Right Answer for the Right Reasons in Global Land Carbon Sink Estimates
Source: Glob Chang Biol. 2026 Apr 7;32(4):e70840. doi: 10.1111/gcb.70840 (PMC13054780; doi:10.1111/gcb.70840)
Supplement: Supplementary file 1 — Figure S1: Spatial patterns of mean annual net ecosystem exchange (NEE) across 16 global. Figure S2: Spatial patterns of net ecosystem exchange (NEE) trends from representative. Figure S3: Overview of FLUXNET sites used in this study. (a) Spatial distribution of sites overlaid on global climate zones, with symbols indicating IGBP. Figure S4: Comparison of Reco‐GPP relationships derived from four flux partitioning methods. Figure S5: Cross‐site GPP‐Reco relationships based on multi‐year mean values at each. Figure S6: Interannual GPP‐Reco relationships based on year‐to‐year anomalies at each. Figure S7: Cross‐site GPP‐ET relationships based on multi‐year mean values at each. Figure S8: Interannual GPP‐ET relationships based on year‐to‐year anomalies at each. [file GCB-32-e70840-s001.pdf]

Supplementary Information for

Towards right answer for the right reasons in global land carbon sink estimates

Helin Zhang[a], Youngryel Ryu[a,b,c*], Sungchan Jeong [a]

[a]*Research Institute of Agriculture and Life Sciences, Seoul National University, Seoul, Republic of Korea*

[b]*Department of Landscape Architecture and Rural Systems Engineering, Seoul National University, Seoul, Republic of Korea*

[c]*SNU Energy Initiatives, Seoul National University, Seoul, Republic of Korea*

Corresponding author: yryu@snu.ac.kr (Youngryel Ryu)

This PDF file includes:

Supplementary Figure 1 to 8

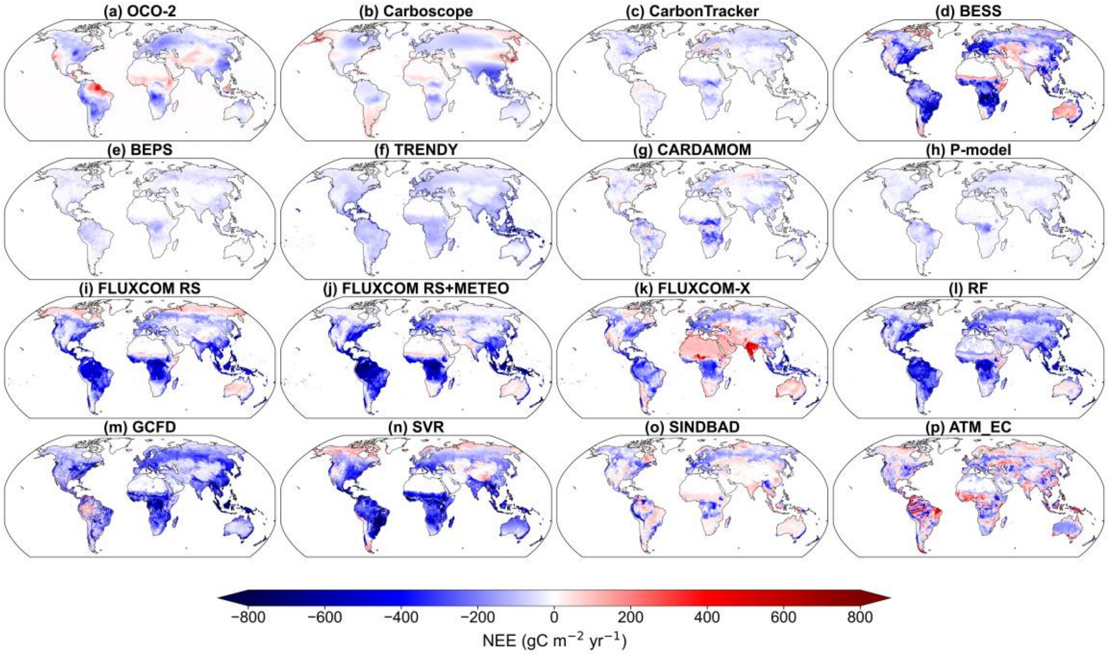

Figure S1 Spatial patterns of mean annual net ecosystem exchange (NEE) across 16 global products. (a–c) Top-down atmospheric inversions, (d–h) Process-based models, (i–n) Machine learning models, and (o–p) Hybrid approaches.

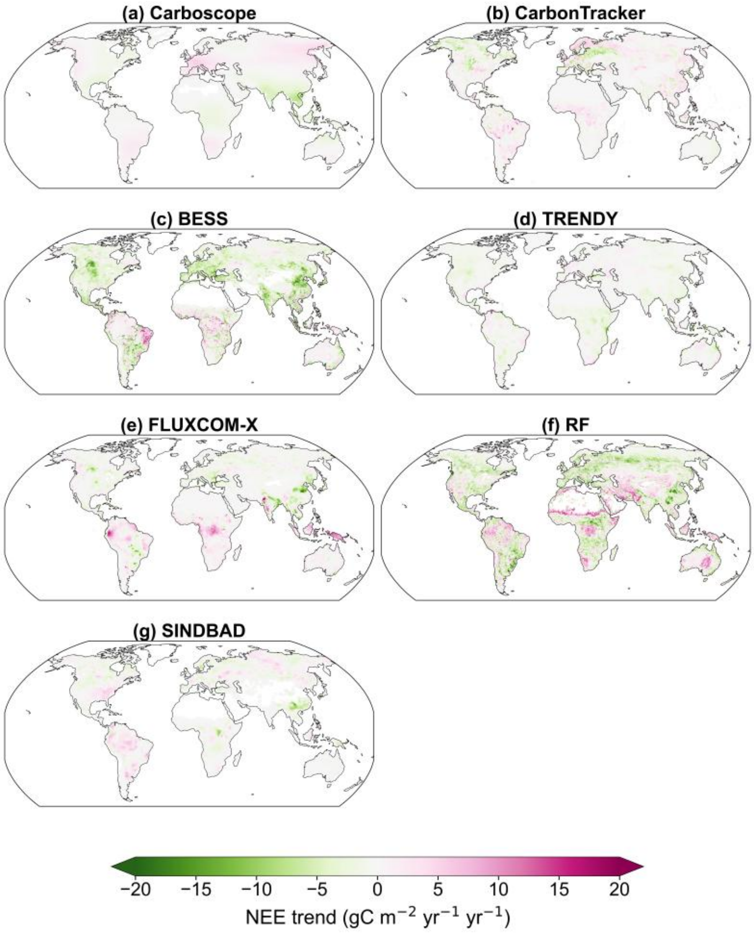

Figure S2 Spatial patterns of net ecosystem exchange (NEE) trends from representative products of each methodological approach (2001–2019). (a–b) Top-down atmospheric inversions, (c–d) Process-based models, (e–f) Machine learning models, and (g) Hybrid approach.

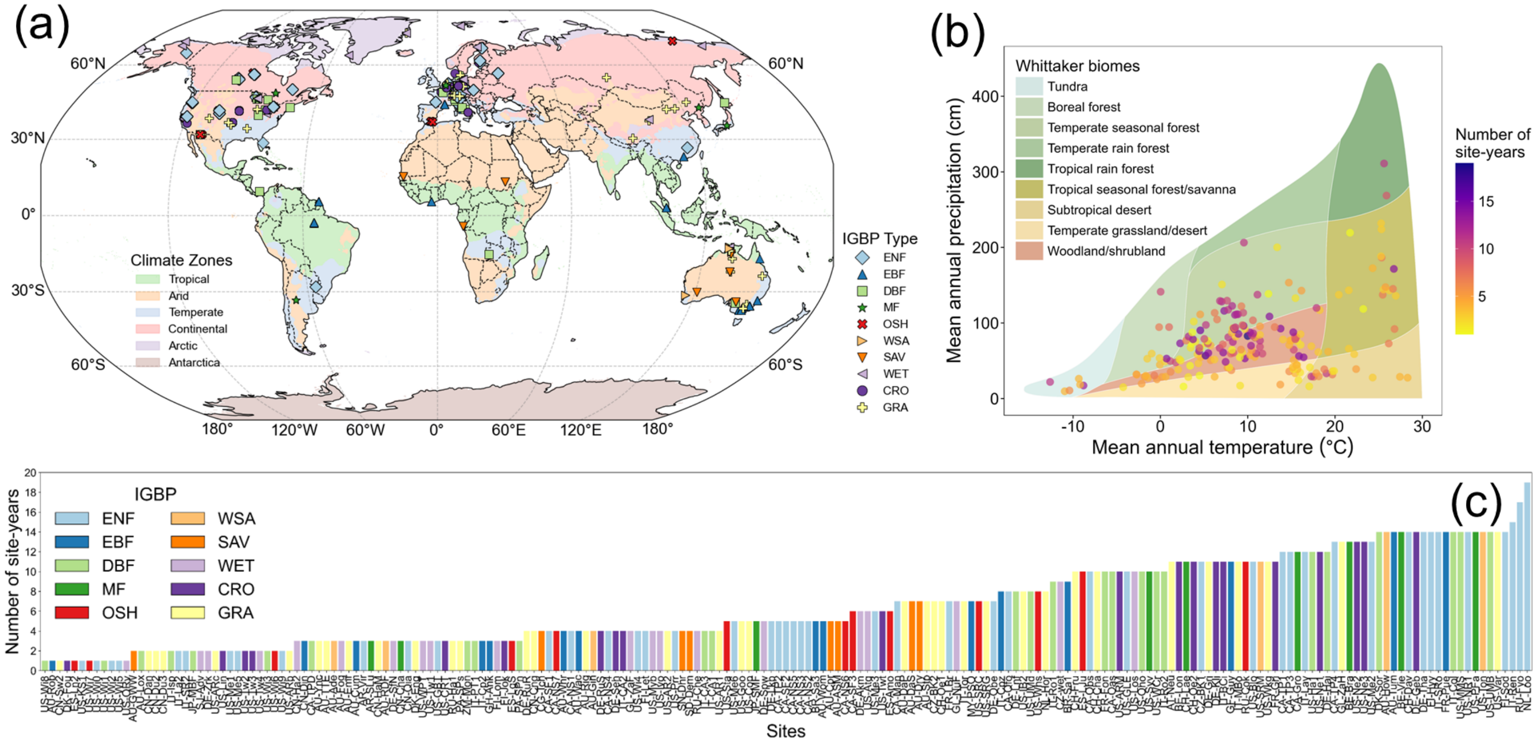

Figure S3 Overview of FLUXNET sites used in this study. (a) Spatial distribution of sites overlaid on global climate zones, with symbols indicating IGBP vegetation types. (b) Site distribution across Whittaker biomes in mean annual temperature-precipitation space, with colors indicating the number of site-years at each location. (c) Number of site-years for each site, colored by IGBP vegetation type.

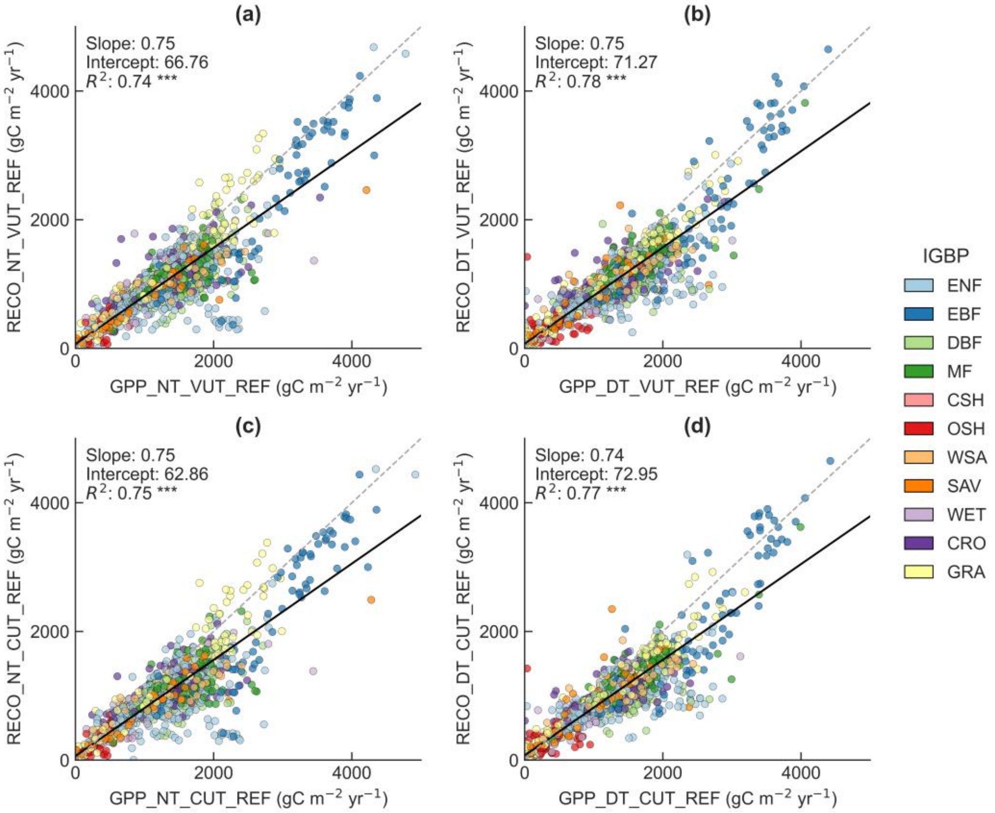

Figure S4 Comparison of R_{eco}-GPP relationships derived from four flux partitioning methods. (a) NT_VUT_REF, (b) DT_VUT_REF, (c) NT_CUT_REF, and (d) DT_CUT_REF.

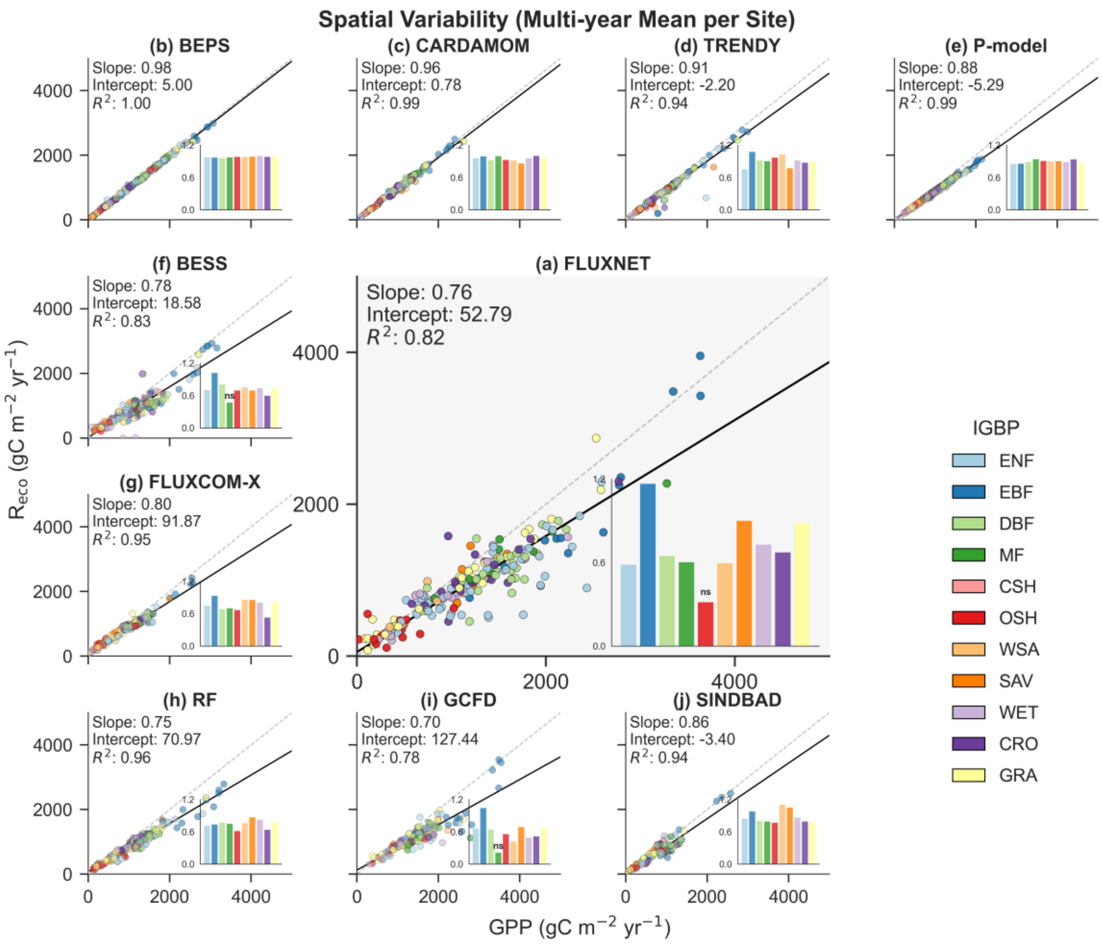

Figure S5 Cross-site GPP-R_{eco} relationships based on multi-year mean values at each FLUXNET site. Inset bar charts show the regression slopes for individual IGBP vegetation types.

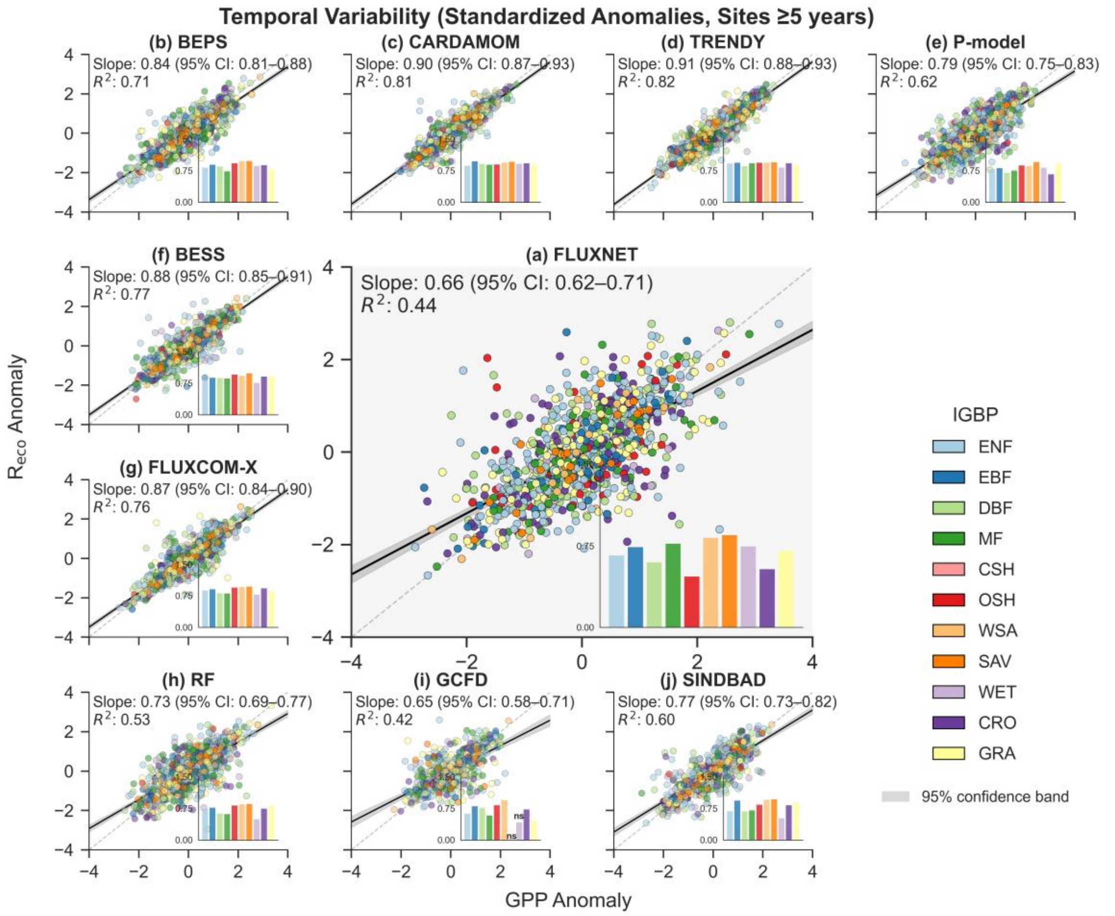

Figure S6 Interannual GPP-R_{eco} relationships based on year-to-year anomalies at each FLUXNET site. Anomalies were calculated by subtracting the site-specific multi-year mean and dividing by the site-specific standard deviation for each variable. Only sites with at least five years of observations were included. Inset bar charts show the regression slopes for individual IGBP vegetation types.

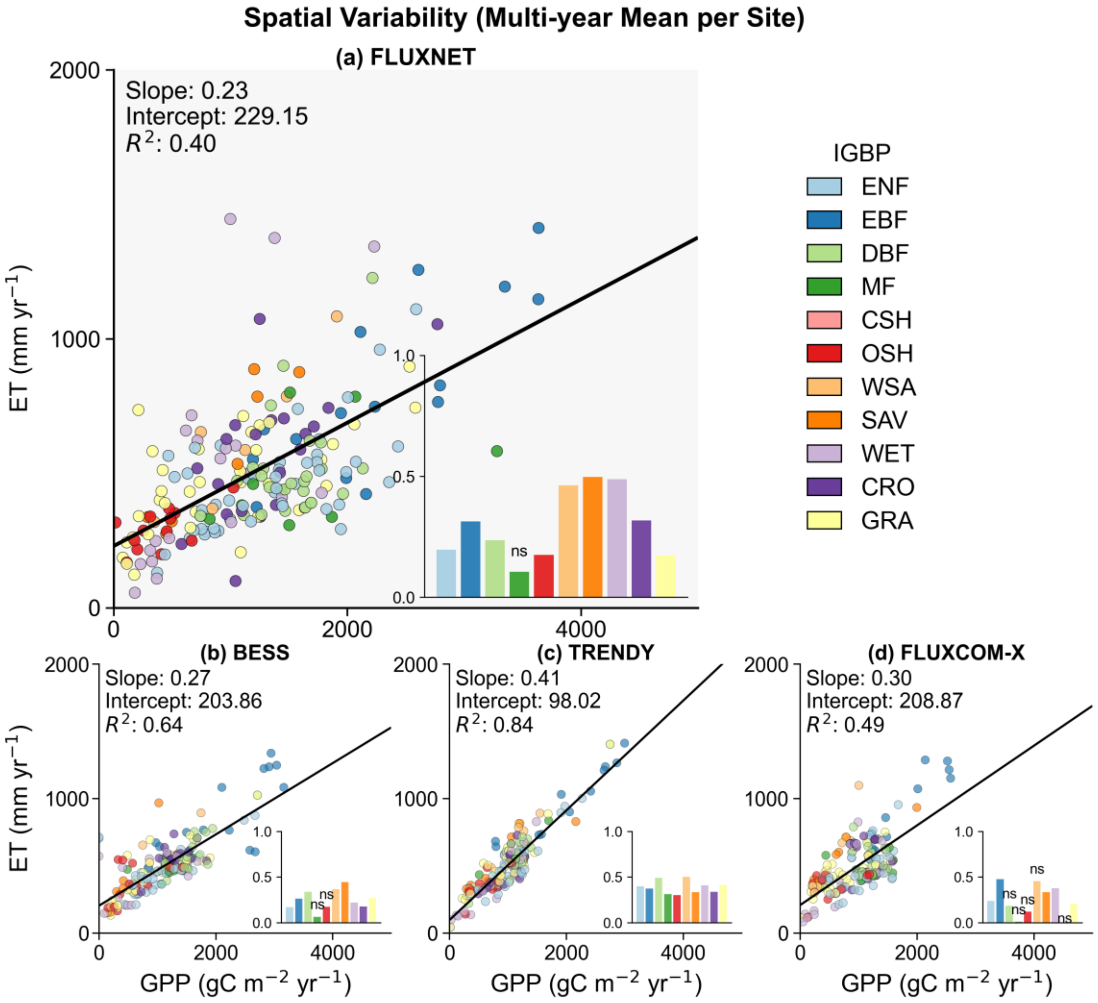

Figure S7 Cross-site GPP-ET relationships based on multi-year mean values at each FLUXNET site. Inset bar charts show the regression slopes for individual IGBP vegetation types.

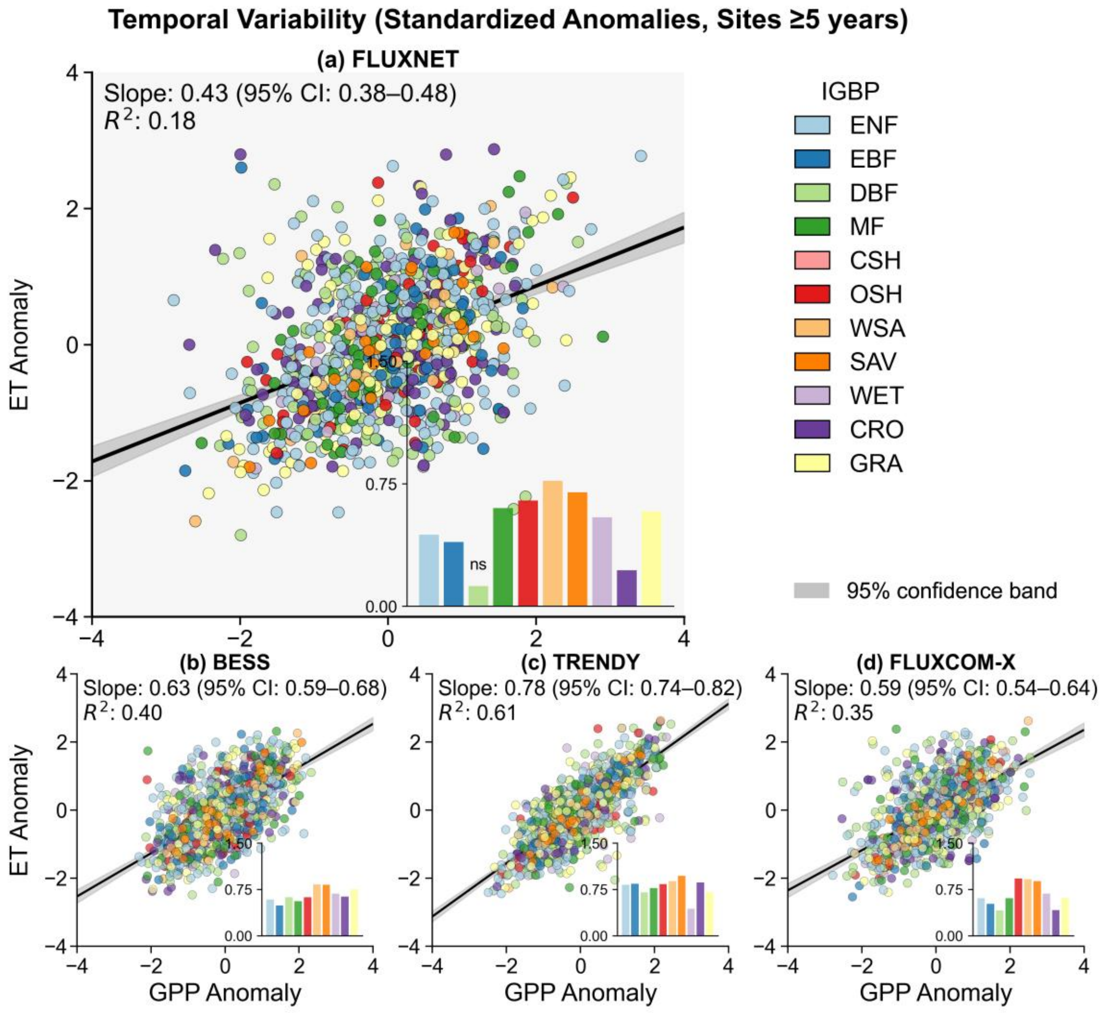

Figure S8 Interannual GPP-ET relationships based on year-to-year anomalies at each FLUXNET site. Anomalies were calculated by subtracting the site-specific multi-year mean and dividing by the site-specific standard deviation for each variable. Only sites with at least five years of observations were included. Inset bar charts show the regression slopes for individual IGBP vegetation types.
